# Water uptake behaviour of semiconducting glasses formed from hybrid organic–inorganic perovskites

**DOI:** 10.1039/d5cc02507a

**Published:** 2025-09-15

**Authors:** Jay McCarron, Libbie Hardy, Olivia Dunlop, Laura L. J. Whitfield, Isaiah Borne, Bethan Turner, Sam D. Harding, Hongjun Niu, Lauren N. McHugh

**Affiliations:** a Department of Chemistry, University of Liverpool Crown Street Liverpool L69 7ZD UK L.N.Mchugh@liverpool.ac.uk

## Abstract

Here we present the uptake behaviour of mechanochemically synthesised crystalline HOIPs, of the form [TAlA][M(dca)_3_] (TAlA = tetraalkylammonium, M = Mn^2+^ and Fe^2+^, dca = [C_2_N_3_^−^] (dicyanamide)), and their melt-quenched glassy derivatives. The semiconducting nature of these materials, alongside their response to environmentally relevant stimuli, was explored using optoelectronic techniques alongside electrochemical analyses.

## Abstract 2

Glasses formed from HOIPs are shown to exhibit high moisture stability alongside improved adsorption properties. Their semiconducting character positions them as promising candidates for optolectronic applications.

## Graphical abstract

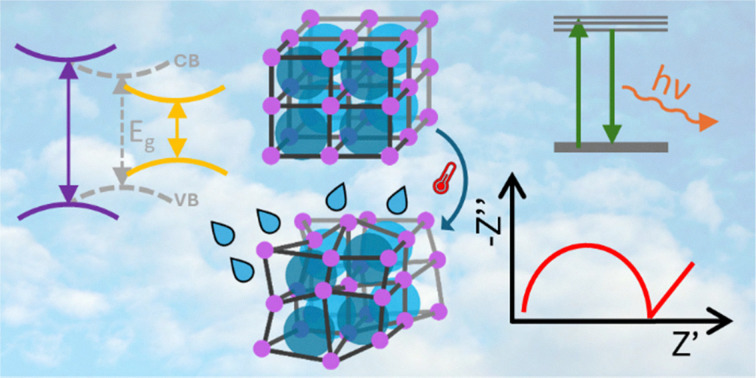

Hybrid organic–inorganic perovskites (HOIPs), of the form ABX_3_ (A = organic cation, B = metallic cation and X = anion) (Fig. 1), have emerged, in recent years, at the forefront of materials discovery. With high structural diversity, several systems have emerged employing chemically distinct ‘X’ site ionic building blocks giving rise to sub-families such as formates [HCOO^−^],^1^ dicyanamides [C_2_N_3_^−^],^2^ and azides [N_3_^−^].^3^ Similarly, diverse structure types can arise from the variation of ‘A’ sites, such as the tetraalkyl ammoniums (TAlA) [(C_*n*_H_2*n*+1_)_4_N^+^], where upon increasing the alkyl chain length the structure type adopted transitions to accommodate the additional steric bulk (Fig. 1).^4^ The chemical versatility afforded to these crystalline systems has facilitated increasing levels of investigation for advanced applications such as highly responsive barocaloric materials.^5^ The growing interest in HOIPs is unsurprising with the recent demonstration that, structurally similar, hybrid perovskites are challenging conventional photovoltaic materials such as silicon in a range of application fields including solar cells.^6^ This places hybrid perovskites amongst the most promising technologies to drive the sustainable revolution. However, current perovskite systems are known to lack stability, with atmospheric moisture known to commonly lead to degradation and significant reductions in general performances.^7^ The highly tuneable diverse structural nature of HOIPs allows for them to be designed to overcome the drawbacks associated with other perovskite materials. With the recent reporting of melt-quenching in dicyanamide HOIPs, in the range of 100–260 °C, to produce glasses, a great deal of interest has emerged in understanding both the underlying chemistries and the bulk properties of these materials.^8^ It has been shown that, through the careful selection of ‘A’ and ‘B’ sites, fine control over thermal properties such as: melting onset temperature (*T*_m_), glass transition temperature (*T*_g_) and decomposition temperature (*T*_d_), can be readily achieved.^9^ Although in their relative infancy, glasses derived from HOIPs have shown intriguing property changes with respect to their parent crystalline materials. Where [TAlA][M(dca)_3_] are readily soluble in water (H_2_O) and decompose upon exposure to both acidic and basic mediums, the melt-quenched glasses formed from these materials have shown no solubility in water and high resistance to degradation across a wide pH range (2–10).^10^ Additionally, bulk properties such as host–guest interactions remain under-explored, an area seeing significant investigation in other hybrid framework materials.^11^ Understanding, and exploiting, these property changes will be key to seeing the broader implementation of this class of materials in real world applications.

**Fig. 1:**
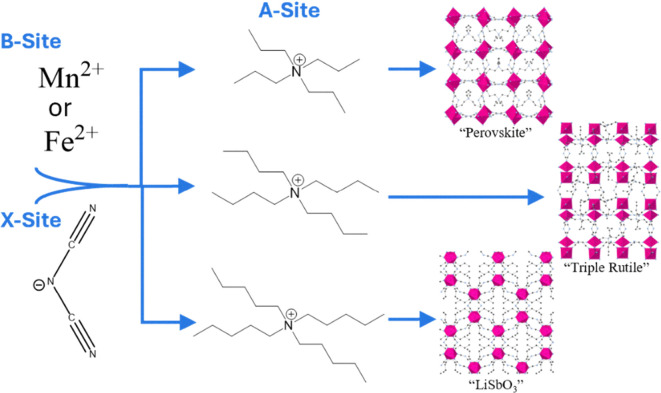
Representation of dicyanamide HOIP structural components and the resulting structure types produced by employing varying A-sites. Tetrapropyl ammonium, tetrabutyl ammonium and tetrapentyl ammonium produce perovskite, triple rutile and LiSbO_3_ structure types, respectively. Crystal structures adapted using data in Ref. 4.

The wider investigation of HOIPs has been hindered, in part, by the time-consuming slow evaporation techniques employed for their syntheses. Often employing large quantities of organic solvents at high temperatures for prolonged periods, typical syntheses are environmentally unfavourable. Mechanochemical synthesis, shown recently to facilitate rapid and scalable syntheses of several hybrid materials, has emerged at the forefront of sustainable synthetic methodology by directly tackling a number of issues previously identified.^10^

Here, we expand upon the reports of increased carbon dioxide (CO_2_) uptake upon formation of the glassy states of a series of HOIPs. We also investigate the responses of HOIPs to water vapour both in terms of uptake behaviour and associated stability concerns. Importantly, we highlight the differing semiconducting behaviour of these systems in the crystalline, glassy and glasses after exposure to water vapour. With the recent demonstration that mechanosynthesis can be utilised for the successful synthesis of dicyanamide HOIPs,^10^ we expand the library of compounds for which this technique can be employed by investigating systems with varying ‘A’ and ‘B’ sites. The previously optimised synthetic procedure for the large scale synthesis of [TPrA][Mn(dca)_3_]^10^ (**TPrAMn**) was found to be readily transferable and was employed in the synthesis of [TPrA][Fe(dca)_3_] (**TPrAFe**), [TBuA][Mn(dca)_3_] (**TBuAMn**), [TBuA][Fe(dca)_3_] (**TBuAFe**), [TPnA][Mn(dca)_3_] (**TPnAMn**) and [TPnA][Fe(dca)_3_] (**TPnAFe**), which until now have only been synthesised by conventional solvothermal based routes.^9^ These rapid syntheses repeatedly yielded phase pure, highly crystalline, materials (confirmed by PXRD analysis, Fig. S1–S6).

These materials were probed using thermogravimetric analysis (TGA) (Fig. S7–S9) and differential scanning calorimetry (DSC) (Fig. S10–S15) to determine if their thermal properties are tied to synthetic method. The *T*_d_ and *T*_m_ of the mechanochemically synthesised materials were found to be in good agreement with those previously reported suggesting that thermal characteristics are not coupled to synthetic route.^9^ Quenching of the melt states of these materials was found to produce glasses (denoted with the prefix ‘a_g_’), displaying clear *T*_g_ and amorphous diffraction patterns in all of the materials, except **TPnAFe** which did not have a clear *T*_g_. The incredibly fine glass transition in this material has been reported previously,^9^ and as such, the melt-quenched product of **TPnAFe** will be considered a glass.

CO_2_ isotherms were collected for the crystalline and glassy states of **TPrAMn**, **TPrAFe**, **TBuAMn**, **TBuAFe**, **TPnAMn** and **TPnAFe** to investigate the impact of ‘A’ and ‘B’ site substitution on the uptake behaviour of these materials (Full isotherms are shown in Fig. S16a–f) with the maximum uptakes for each material summarised in Fig. 2. Across all 6 materials, uptakes increase for the melt-quenched products, with **a**_**g**_**-TPrAMn**, **a**_**g**_**-TPrAFe**, **a**_**g**_**-TBuAFe**, **a**_**g**_**-TPnAMn** and **a**_**g**_**-TPnAFe** showing *c.a*. 3 to 3.5-fold increases and **a**_**g**_**-TBuAMn** showing a 9-fold increase in maximum uptake relative to their crystalline precursors. The observed increase in CO_2_ uptake, upon glass formation, is likely caused by significant changes in particle shape and size. Upon melt-quenching, the materials undergo significant bulk morphological changes as they transform from agglomerates of small, rounded particles to larger fused pieces presenting extensive surface cavities (Fig. S17a–r). We hypothesise that these cavities increase the overall area for analyte/material interactions to take place, in turn increasing the measured uptake. Additionally, the hysteresis observed in the isotherms collected in the glasses can be attributed to non-genuine kinetic induced outgassing conditions.^12^ Interestingly, it appears as though the ‘B’ site employed has little impact on the observed surface/gas interaction with only minimal changes observed upon variation. However, it does appear that the ‘A’ site utilised has a more pronounced effect with the systems employing TPrA^+^ showing maximum uptakes approximately 2.5 times higher than the systems employing TBuA^+^ or TPnA^+^. This suggests that, in conjunction with the morphological changes, intrinsic structural effects are influencing the uptake of CO_2_, although only in a limited capacity. We suggest that the shorter alkyl chains of TPrA^+^ offer greater void space within the M(dca)_3_ framework than the sterically bulkier TBuA^+^ and TPnA^+^, facilitating easier ingress of CO_2_ within the material.

**Fig. 2:**
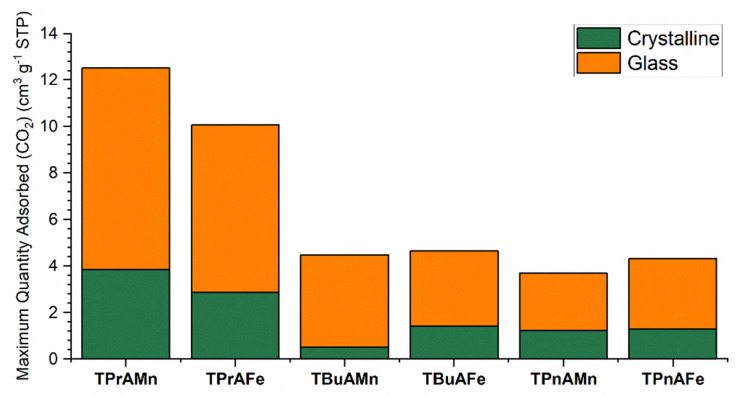
Maximum CO_2_ adsorbed for the crystalline (green) and glassy (orange) states of TPrAMn, TPrAFe, TBuAMn, TBuAFe, TPnAMn, TPnAFe.

With measurable uptake of CO_2_ occurring in these materials, we sought to investigate host–guest interactions of other species. Water vapour was considered an ideal candidate as, previously highlighted, perovskite solar cells generally degrade upon exposure to atmospheric moisture. The crystalline phases of the HOIPs were exposed to a 95% relative humidity (% RH) environment for one week to investigate their stability in high levels of water vapour. Analysis of the materials pre and post-exposure to water vapour by powder *x*-ray diffraction (PXRD) and FTIR showed no crystallographic, structural or bonding changes occurring between the pre and post-exposure states (Fig. 3). Alongside these analyses, CHN elemental analysis remained virtually unchained with only minute differences in composition detected (Table S2). These methods, in combination, indicate a high level of moisture stability, typically uncommon for hybrid perovskite materials.

**Fig. 3:**
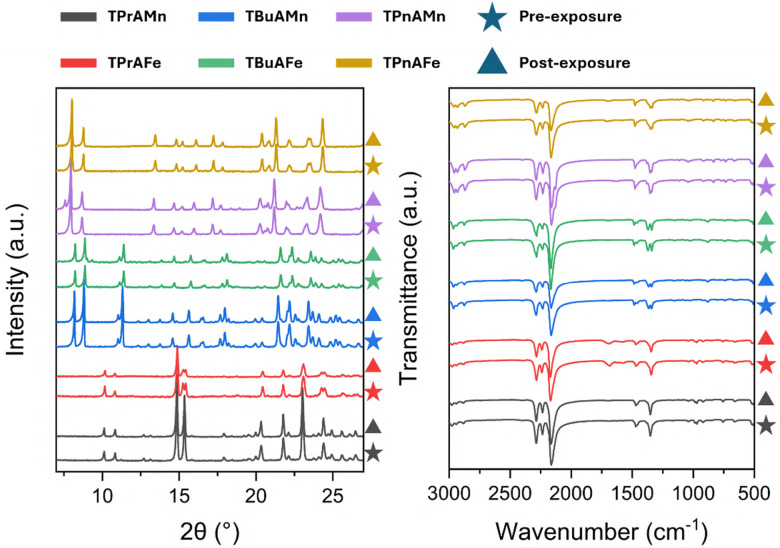
PXRD (left) and FTIR (right) analysis of Pre water vapour exposure (Pre-WV)(Star) and Post water vapour exposure (Post-WV)(Triangle) for TPrAMn (grey), TPrAFe (red), TBuAMn (blue), TBuAFe (green), TPnAMn (purple) and TPnAFe (gold). An enlarged version has been provided in the SI (Fig. S18).

Water adsorption isotherms were measured for all crystalline and glassy systems from 0 to 0.98 relative pressure, equivalent to % RH (Full isotherms available, Fig. S19a–f). Maximum uptake (wt%) at 98% RH are collated for all systems in Fig. 4. Again, the transformation of these materials from crystalline to glass leads to an increased uptake, with maximum water uptake increasing between 2–20 wt% across the series of materials. Although appearing to show a higher affinity toward water, due to the increased uptake at lower pressures, the glasses are more hydrophobic than the crystalline states of these materials, as shown by an increase in water contact angle (Fig. S20 and S21). This observed change in hydrophobicity is possibly driven by the formation of tricyanomelaminate units through the temperature induced trimerisation of dicyanamide ions, confirmed by the appearance of IR peaks at 1653 and 804 cm^−1^ (see Fig. S22 for additional details). With the glasses showing a reduced attraction to water, the increased uptake is again likely influenced by the formation of the cavities in the fused glasses. Additionally, we also believe that sufficient void space is present within the materials to allow penetration of water molecules into the framework structure. As the maximum water uptake decreases across the series with increased alkyl chain length (Fig. 4), we believe that the water molecules accessing the void spaces in the M(dca)_3_ framework are competing with the alkyl ammonium cations and as the size of the cations increases the overall available void space decreases. As such the maximum uptake is limited by the increasingly larger TBuA^+^ and TPnA^+^ cations. The degree of penetration of the analyte species occurs to a greater degree here for water than for CO_2_ due to the smaller kinetic diameter of the molecule (H_2_O = 265 pm, CO_2_ = 330 pm) which allows it to more readily access the void spaces throughout the framework, in turn, enabling it to contribute significantly to the measured water uptake character of these materials. This effect, in combination with the surface cavities formed, leads to the appreciable water uptakes observed. Additionally, the metal ion employed also appears to have an impact on the maximum water uptake in the glasses, with the Fe^2+^ systems all showing higher uptakes that their Mn^2+^ counterparts using the same ‘A’ site cations. Another interesting feature observed for these materials is the significant changes in the profiles of the measured isotherms for the crystalline and glassy states. For the crystalline materials, closed hysteresis loops are observed (where closing occurs between 50–70% RH) suggesting a standard H3 type hysteresis and a slit-shaped pore structure undergoing capillary condensation behaviour, commonly observed for aggregates, resembling those seen in the crystalline HOIPs (Fig. S17a–r).^13^ In contrast to this, the isotherms for the glass states all show significant hysteresis in which the loop does not close. With no morphological changes observed in the glasses after exposure to water vapour (as confirmed by SEM, Fig. S17a–r), we suggest that surface clustering of adsorbed water leads to the observed hysteresis as opposed to a conventional capillary condensation model.^14^

**Fig. 4:**
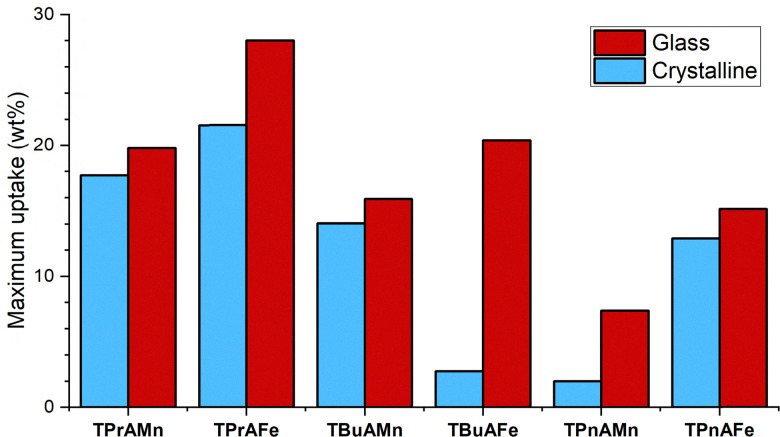
Maximum wt% of water uptake achieved for crystalline (blue) and glass (red) TPrAMn, TPrAFe, TBuAMn, TBuAFe, TPnAMn, TPnAFe.

With both the crystalline and glassy phases of these materials displaying water uptake behaviour, we sought to investigate if the optoelectronic properties of these systems would be significantly impacted by this process. UV-Vis and fluorescence spectroscopies were utilised to investigate the optoelectronic properties of the crystalline and glass states, and the glass post exposure to water vapour. Significant changes in UV-Vis reflectance profiles were measured between the crystalline and glassy phases of these materials emphasising the significant structural changes occurring during the melt-quench procedure (Fig. S23a–f). The optically allowed direct bandgaps were determined for each system (Fig. 5) using the Kubelka–Munk method with *x*-intercept of the first linear region taken as the optical bandgap (Fig. S24–S26), with differences clearly observed between the material states. The measured bandgaps for the crystalline states were found to be in the 2.1–2.7 eV range, positioning these materials as semiconductors. Upon melt quenching, several changes are observed in the measured bandgap values. The measured bandgaps of **a**_**g**_**-TPrAMn** and **a**_**g**_**-TBuAMn** show increases of 0.4 and 0.3 eV respectively, in comparison to their crystalline precursors. This increase is likely caused by an alteration in the coordination environment of metal ion sites, a phenomenon observed in the crystalline to amorphous transition of other materials.^15^ In contrast, **TPrAFe**, **TBuAFe**, **TPnAMn** and **TPnAFe** all show decreases in the measured bandgaps upon glass formation. A gap reduction is observed frequently for amorphous materials, due to the emergence in mid gap defects caused by the loss of long-range structural order.^16^ The emergence of mid gap defects in these materials is supported by the observed Urbach tail in the Kubelka–Munk curves (Fig. S24–S26). Interestingly, upon prolonged exposure to water vapour (95%RH, 1 week) the TPrA^+^ and TBuA^+^ materials show minimal change in bandgap, further emphasising the high levels of stability displayed by these materials. Bandgap changes are, however, observed for the TPnA^+^ based systems with **a**_**g**_**-TPnAMn** showing a decrease of approximately 0.5 eV and **a**_**g**_**-TPnAFe** an increase of 0.3 eV. This clearly quantifiable change in bandgap coupled with the lack of degradation suggest that these specific systems could potentially be utilised in moisture sensing applications. Room temperature photoluminescence (PL) data was collected for the crystalline, glass and water vapour exposed glasses (emission spectra are shown in Fig. S27) to further explore the optoelectronic properties of these systems. Direct bandgaps were calculated by converting the maximum emission wavelength to photon energy, representing the relaxation energy of excited electrons back to the valence band. From this, it was found that these materials all displayed an energy gap in the 2.8–3.0 eV range (Fig. 5), again, marking these systems as semiconductors. Electrical conductivities of the crystalline, glassy and glasses after water vapour exposure were measured using alternating current (AC) electrical impedance spectroscopy (EIS) and direct current (DC) methods, with selected examples highlighted in Fig. 5. (Full data can be found in Fig. S28–S31). Calculated room temperature conductivities were found to be in the range of 2 × 10^−4^ – 8.5 × 10^−9^ S cm^−1^ using EIS and 1 × 10^−6^ – 1.5 × 10^−13^ S cm^−1^ using DC methods (Table S1), again marking these materials as semiconductors.^17^ Although melt-quenching is commonly expected to reduce grain boundary contributions and subsequently increase electrical conductivities,^18^ it was found that, upon glass formation, these systems show a decrease in conductivity. This is likely caused by the loss of the defined pathways present in crystalline materials leading to formation of charge carrier traps in the amorphous state.^19^ Additionally, exposure to water vapour appears to either have little impact on the conductivity of the glass, or leads to further decreases in conductivity.

**Fig. 5:**
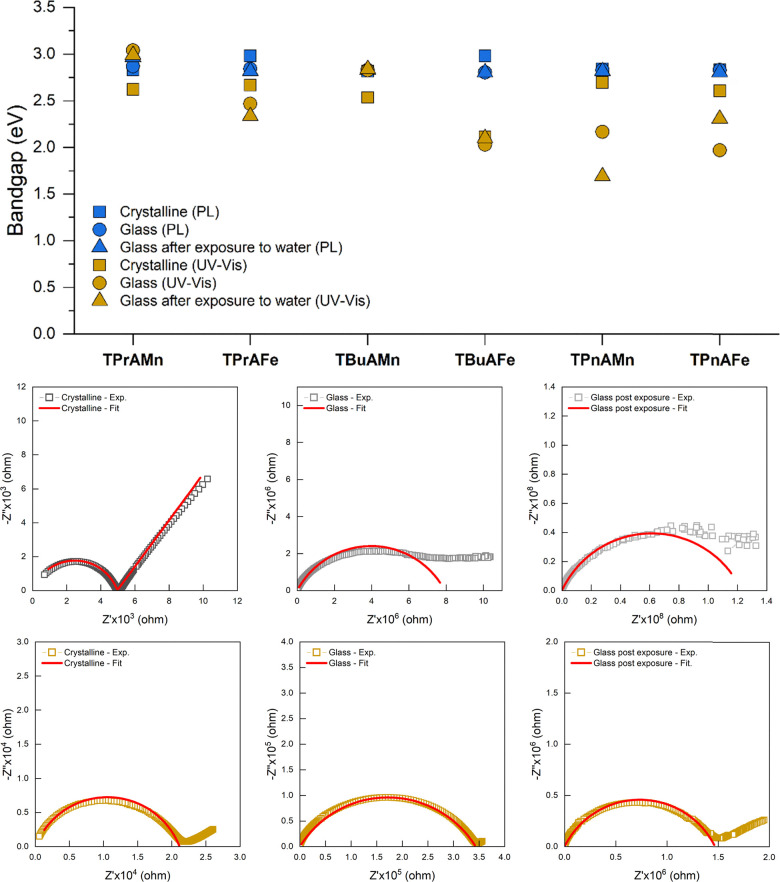
Top; optical bandgaps measured using photoluminescence (blue) and UV-Vis (gold) for the crystalline, glass, and glass post water vapour exposure. Bottom; nyquist plots for the crystalline, glass, and glass post water vapour exposure states of TPrAMn (grey) and TPnAFe (gold) showing increased resistance upon glass formation and a further increase post exposure to water vapour. Equivalent circuit fits are shown as red lines.

In conclusion, we have presented an expanded library of highly-tuneable, and sustainably synthesised glass forming HOIPs, showing appreciable levels of uptake of both CO_2_ and water vapour. Additionally, we highlight the resistance of the crystalline phases of these materials to high levels of relative humidity, a property uncommon for other semiconducting perovskites. The measured optical bandgaps of these materials in both the crystalline and glassy states positions them as semiconducting materials. Additionally, AC impedance and DC current–voltage measurements show a wide range of electrical conductivities, including a reduction in measured conductivity upon glass formation, with respect to the parent crystalline states. The high stability of these HOIPs coupled with their highly tuneable nature, and the subsequent influence on material properties such as guest species uptake, optoelectronic and electrochemical properties, positions these new emerging materials at the forefront of discovery in the energy landscape.

LNM; conceptualization, funding acquisition, project administration, supervision, writing – original draft, review and editing. JM; investigation, formal analysis, visualization, writing – original draft, review and editing. LH, OD, LLJW, IB, BT, SDH and HN; investigation, writing – review and editing.

LNM and JM wish to thank the University of Liverpool and BT thanks the Faculty of Science and Engineering at the University of Liverpool for PhD project funding. LH thanks the Henry Royce Institute for funding through the Royce undergraduate internship scheme. This project has received funding from the European Research Council (ERC) under the European Union's Horizon 2020 research and innovation program (Grant agreement No. 856405). LLJW and HN thank the Engineering and Physical Sciences Research Council (EPSRC) for funding under EP/V026887. The authors wish to thank Prof. Andrew I. Cooper and Prof. Matthew J. Rosseinsky for access to equipment and resources.

## Conflicts of interest

There are no conflicts to declare.

## Supplementary Material

CC-061-D5CC02507A-s001

## Data Availability

The data supporting this article have been included as part of the SI. The experimental methods and characterisation data (structural, thermal, adsorption, microscopy, optoelectronic and electrochemical) supporting this article have been included in the SI. See DOI: https://doi.org/10.1039/d5cc02507a.
