# Supplementary material for: Water uptake behaviour of semiconducting glasses formed from hybrid organic–inorganic perovskites
Source: Chem Commun (Camb). 2025 Sep 15;61(84):16404–7. doi: 10.1039/d5cc02507a (PMC12455473; doi:10.1039/d5cc02507a)
Supplement: CC-061-D5CC02507A-s001 [file CC-061-D5CC02507A-s001.pdf]

Supplementary Information (SI) for ChemComm.
This journal is © The Royal Society of Chemistry 2025

Supporting Information

Synthesis and characterisation 2
Structural Refinements 5
Thermogravimetric analysis (TGA) 10
Differential scanning calorimetry 13
CO_2 adsorption and desorption isotherms 16
Scanning Electron Microscopy 17
Water vapour stability 18
Water sorption isotherms 19
Water contact angle measurements 20
FTIR 21
UV-Vis reflectance data 22
Band gap calculations using the Kubelka-Munk function 23
Photoluminescence spectroscopy – Emission Spectra 26
Conductivity measurements 27
CHN elemental analysis 32
References 32

Synthesis and characterisation

Synthesis

Methods have been adapted from previously published methods.[1] All syntheses were performed mechanochemically using a RETSCH Mixer Mill MM 400.

[TPrA][Mn(dca)$_3$] Tetrapropyl ammonium bromide (439 mg, 1.65 mmol), manganese chloride (207.5 mg, 1.65 mmol), sodium dicyanamide (445.2 mg, 5 mmol) and a 2:1 water: ethanol mix (75 µL) were added to a 50 mL stainless steel ball mill jar followed by 2 x 20 mm stainless steel balls. The contents were milled (30 Hz, 30 min), the resulting grey powder was washed quickly with ice cold water (2 x 30 mL) and was dried in vacuo.

[TPrA][Fe(dca)$_3$] Tetrapropyl ammonium bromide (439 mg, 1.65 mmol), iron chloride (266.5 mg, 1.65 mmol), sodium dicyanamide (445.2 mg, 5 mmol) and a 2:1 water: ethanol mix (75 µL) were added to a 50 mL stainless steel ball mill jar followed by 2 x 20 mm stainless steel balls. The contents were milled (30 Hz, 30 min), the resulting orange powder was washed quickly with ice cold water (2 x 30 mL) and was dried in vacuo.

[TBuA][Mn(dca)$_3$] Tetrabutyl ammonium bromide (531.9 mg, 1.65 mmol), manganese chloride (207.5 mg, 1.65 mmol), sodium dicyanamide (445.2 mg, 5.00 mmol) and a 2:1 water: ethanol mix (125 µL) were added to a 50 mL stainless steel ball mill jar followed by 2 x 20 mm stainless steel balls. The contents were milled (30 Hz, 30 min), the resulting grey powder was washed quickly with ice cold water (2 x 30 mL) and was dried in vacuo.

[TBuA][Fe(dca)$_3$] Tetrabutyl ammonium bromide (531.9 mg, 1.65 mmol), iron chloride (266.5 mg, 1.65 mmol), sodium dicyanamide (445.2 mg, 5.00 mmol) and a 2:1 water: ethanol mix (125 µL) were added to a 50 mL stainless steel ball mill jar followed by 2 x 20 mm stainless steel balls. The contents were milled (30 Hz, 30 min), the resulting orange powder was washed quickly with ice cold water (2 x 30 mL) and was dried in vacuo.

[TPnA][Mn(dca)$_3$] Tetrapentyl ammonium bromide (809.7 mg, 1.65 mmol), manganese chloride (207.5 mg, 1.6 mmol), sodium dicyanamide (445.2 mg, 5.00 mmol) and a 2:1 water: ethanol mix (125 µL) were added to a 50 mL stainless steel ball mill jar followed by 2 x 20 mm stainless steel balls. The contents were milled (30 Hz, 30 min), the resulting grey powder was washed quickly with ice cold water (2 x 30 mL) and was dried in vacuo.

[TPnA][Fe(dca)$_3$] Tetrapentyl ammonium bromide (809.7 mg, 1.65 mmol), iron chloride (267.5 mg, 1.65 mmol), sodium dicyanamide (445.2 mg, 5.00 mmol) and a 2:1 water: ethanol mix (125 µL) were added to a 50 mL stainless steel ball mill jar followed by 2 x 20 mm stainless steel balls. The contents were milled (30 Hz, 30 min), the resulting orange powder was washed quickly with ice cold water (2 x 30 mL) and was dried in vacuo.

Glasses were formed by the melt-quenching, under an inert atmosphere, of the crystalline phase of each material in both a differential scanning calorimeter and tube furnace, thermal procedures were the same for both pieces of equipment. Powder crystalline materials was equilibrated at 25 °C and heated to T_{max} (detailed below for each material) at a rate of 10 °C min^{-1}. The samples were held at T_{max} for 1 minute before being cooled to 25 °C at a rate of 3 °C min^{-1}.

T_{max}; [TPrA][Mn(dca)$_3$] = 277.5 °C, [TPrA][Fe(dca)$_3$] = 260 °C, [TBuA][Mn(dca)$_3$] = 290 °C, [TBuA][Fe(dca)$_3$] = 270 °C, [TPnA][Mn(dca)$_3$] = 290 °C, [TPnA][Mn(dca)$_3$] = 265 °C.

Characterisation

Powder X-ray Diffraction (PXRD)

Finely ground powder samples were suspended on Mylar sheets in either stainless steel or aluminium well plates and, using a high-throughput XYZ stage, data was collected using a Panalytical Empyrean diffractometer (Cu Kα radiation, $\lambda = 1.5418$ Å) and a PIXcel detector. Data were collected over the range $2° \leq 2\theta \leq 60°$ with a step size of 0.05° over 18 minutes. Data refinement was carried out using TOPAS Academic V7.24 .[2]

Thermogravimetric analysis (TGA)

TGA measurements were performed on a NETZSCH TG 209F1 Libra. Samples (ca. 10 mg) were heated in alumina crucibles from 25–950 °C at a rate of 10 °C min^{-1} under an N_2 atmosphere.

Differential scanning calorimetry (DSC)

DSC measurements were performed on a TA Instruments Discover Differential Scanning Calorimeter. Samples (ca. 6 mg) were placed in hermetically sealed aluminium pans with lids with pinholes. Empty hermetically sealed aluminium pans were used as a reference and were subjected to the same heat/cool cycles as the samples under investigation. All analyses were carried out under an N_2 atmosphere. Data analysis was performed using the Trios software package.[3]

Gas Sorption

The gas uptake measurements and adsorption analysis of CO_2 up to 0.032 p/p° performed on a Micrometric 3-Flex 3500 Gas Sorption Analyser with a micromeritic isocontroller to keep a constant temperature of 0 °C. Samples of *ca.* 90 mg were degassed in situ with nitrogen by heating to 90 °C - 110 °C at a rate of 10 °C min^{-1} under vacuum for 7 hours. Analysis was performed with Micromeretics Flex Version 6.02 software.[4]

Water sorption

Water sorption isotherms were collected from 0–0.98 relative pressure (p/p°) using a Micrometric 3-Flex 3500 Gas Sorption Analyser at 20 °C on approximately 50 mg of sample. Samples were degassed at 100 °C under vacuum. Data was processed using with Micromeretics Flex Version 6.02 software.[4]

UV-Vis spectroscopy

Diffuse-reflectance measurements were collected at room temperature for finely ground powder samples using a Cary 5000 UV-Vis-NIR Spectrophotometer in the range of 200–800 nm.

Photoluminescence spectroscopy

Photoluminescence measurements were collected using an Edinburgh Instruments FLS980-D2S2-STM spectrophotometer, equipped with a 450 W Xe arc lamp, and using excitation and emission monochromators and a photomultiplier tube detector. Measurements were made in air by sandwiching finely ground powder in a quartz cuvette. Photoluminescence spectra were collected using an excitation wavelength of 280 nm, with data collected between 370 and 750 nm using a 2 nm step and a 0.2 s dwell time. A 330 nm filter was employed at the detector to remove unwanted scattering, data was collected across 10 repeats.

Electrical Impedance Spectroscopy

5mm pellets were pressed uniaxially and the surfaces were coated with a thin layer of silver before being mounted in a spring-loaded conductivity sample holder under ambient atmosphere. AC Impedance measurements were then conducted with a Keysight Impedance Analyser E4990A in a frequency range of 20 Hz – 12 MHz with voltage amplitude of 0.1 V. Data was collected at room temperature and fitted with equivalent circuits using the ZView 4 software.

Direct Current conductivity measurements

5mm pellets were pressed uniaxially and the surfaces were coated with a thin layer of silver before being mounted in a spring-loaded conductivity sample holder under ambient atmosphere at room temperature. Data was collected by measuring current in response to a series of applied voltage (1, 1.5, 2, 2.5 and 3 V). Electrical conductivities were extracted by fitting current voltage curves using Ohm's law.

Scanning Electron Microscopy

SEM was performed using a Hitachi S-4800 operated between 3 and 5 kV, collecting backscattered electrons. Samples were affixed to aluminium stubs using carbon tabs and were sputtered with chromium using a Quorum 150V coater.

FTIR

Room temperature Fourier-transformed Infrared spectroscopy was performed on a Bruker Vertex V70 using a diamond ATR unit. Samples were finely ground and measured in the range of 4000 – 400 cm^{-1} at a resolution of 4 cm^{-1} with 32 scans performed.

Structural Refinements

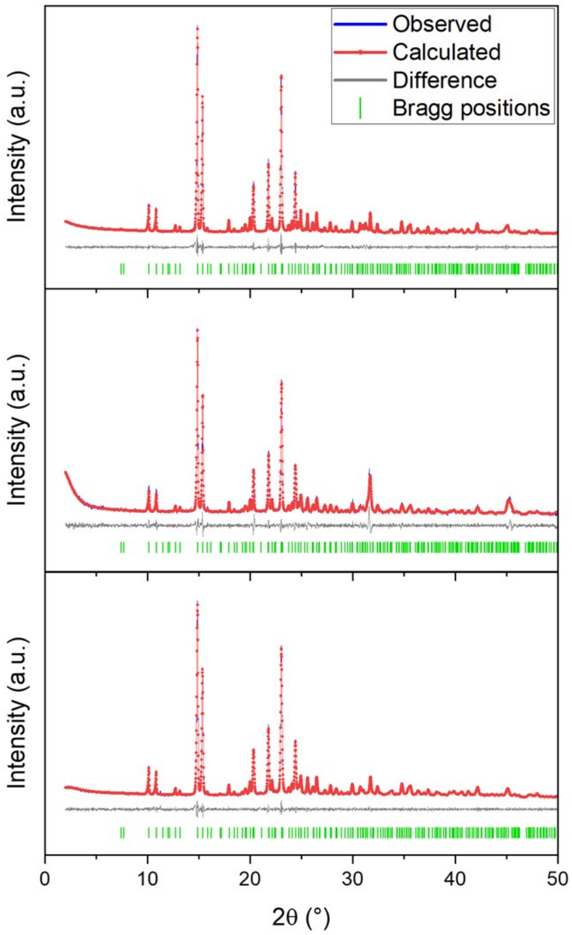

R_{wp}	Space Group	Calculated lattice parameters	Reported lattice parameters[5]
5.41	P-42(1)c	a = 16.2940 (5) Å	a = 16.2945 (4) Å
		b = 16.2940 (5) Å	b = 16.2945 (4) Å
**Structure type**		c = 17.4418 (5) Å	c = 17.4321 (8) Å
		α = 90°	α = 90°
Perovskite		β = 90°	β = 90°
		γ = 90°	γ = 90°

R_{wp}	Space Group	Calculated lattice parameters	Reported lattice parameters[5]
6.46	P-42(1)c	a = 16.2984 (4) Å	a = 16.2945 (4) Å
		b = 16.2984 (4) Å	b = 16.2945 (4) Å
**Structure type**		c = 17.4618 (4) Å	c = 17.4321 (8) Å
		α = 90°	α = 90°
Perovskite		β = 90°	β = 90°
		γ = 90°	γ = 90°

R_{wp}	Space Group	Calculated lattice parameters	Reported lattice parameters[5]
4.16	P-42(1)c	a = 16.2832 (4) Å	a = 16.2945 (4) Å
		b = 16.2832 (4) Å	b = 16.2945 (4) Å
**Structure type**		c = 17.4260 (4) Å	c = 17.4321 (8) Å
		α = 90°	α = 90°
Perovskite		β = 90°	β = 90°
		γ = 90°	γ = 90°

Fig. S1 *Pawley refinements for 3 repeats of the mechanochemically synthesised [TPrA][Mn(dca)$_3$].*

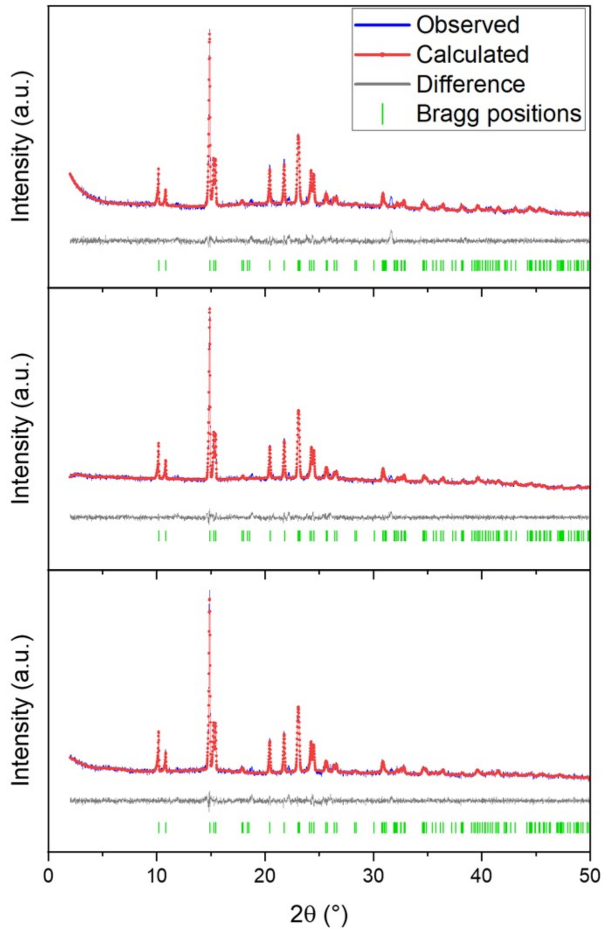

R_{wp}	Space Group	Calculated lattice parameters	Reported lattice parameters[6]
3.80	Ibam	a = 11.4599 (4) Å	a = 11.5219 (4) Å
		b = 11.5996 (4) Å	b = 11.5355 (4) Å
**Structure type**		c = 17.3512 (4) Å	c = 17.3841 (5) Å
		α = 90°	α = 90°
Perovskite		β = 90°	β = 90°
		γ = 90°	γ = 90°

R_{wp}	Space Group	Calculated lattice parameters	Reported lattice parameters[6]
4.08	Ibam	a = 11.4664 (4) Å	a = 11.5219 (4) Å
		b = 11.6078 (4) Å	b = 11.5355 (4) Å
**Structure type**		c = 17.3606 (4) Å	c = 17.3841 (5) Å
		α = 90°	α = 90°
Perovskite		β = 90°	β = 90°
		γ = 90°	γ = 90°

R_{wp}	Space Group	Calculated lattice parameters	Reported lattice parameters[6]
3.19	Ibam	a = 11.4595 (4) Å	a = 11.5219 (4) Å
		b = 11.5878 (4) Å	b = 11.5355 (4) Å
**Structure type**		c = 17.3434 (4) Å	c = 17.3841 (5) Å
		α = 90°	α = 90°
Perovskite		β = 90°	β = 90°
		γ = 90°	γ = 90°

Fig. S2 *Pawley refinements for 3 repeats of the mechanochemically synthesised [TPrA][Fe(dca)3].*

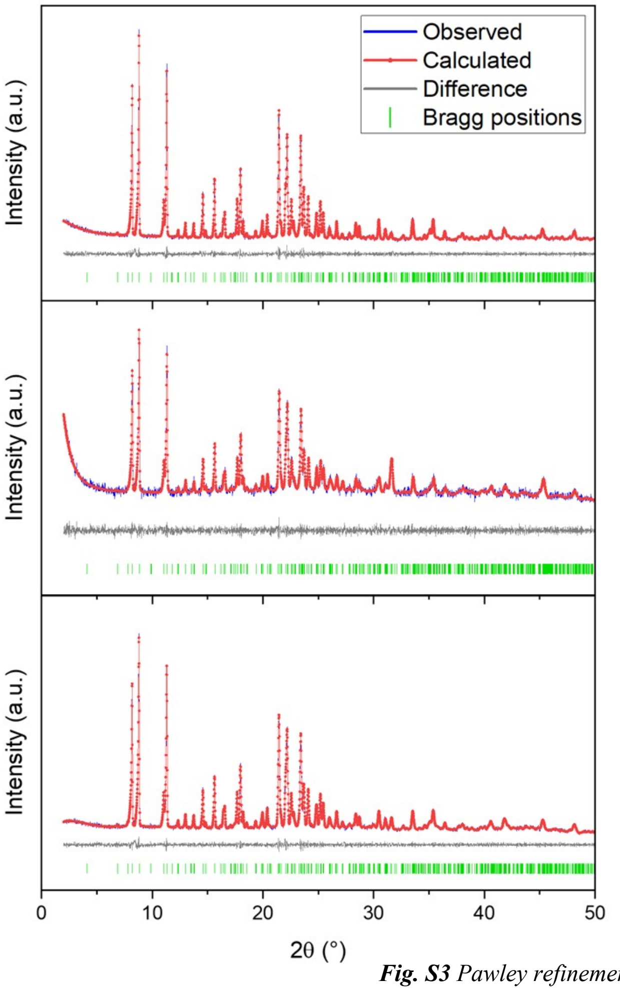

R_{wp}	Space Group	Calculated lattice parameters	Reported lattice parameters[5]
4.86	$P2_12_12$	a = 15.9921 (4) Å	a = 16.0107 (6) Å
		b = 16.0125 (4) Å	b = 16.0114 (6) Å
**Structure type**		c = 21.5445 (4) Å	c = 21.5577 (8) Å
		α = 90°	α = 90°
Triple-Rutile		β = 90°	β = 90°
		γ = 90°	γ = 90°

R_{wp}	Space Group	Calculated lattice parameters	Reported lattice parameters[5]
5.87	$P2_12_12$	a = 15.9937 (4) Å	a = 16.0107 (6) Å
		b = 15.9676 (4) Å	b = 16.0114 (6) Å
**Structure type**		c = 21.5131 (4) Å	c = 21.5577 (8) Å
		α = 90°	α = 90°
Triple-Rutile		β = 90°	β = 90°
		γ = 90°	γ = 90°

R_{wp}	Space Group	Calculated lattice parameters	Reported lattice parameters[5]
4.12	$P2_12_12$	a = 15.9773 (4) Å	a = 16.0107 (6) Å
		b = 16.0040 (4) Å	b = 16.0114 (6) Å
**Structure type**		c = 21.5250 (4) Å	c = 21.5577 (8) Å
		α = 90°	α = 90°
Triple-Rutile		β = 90°	β = 90°
		γ = 90°	γ = 90°

Fig. S3 *Pawley refinements for 3 repeats of the mechanochemically synthesised [TBuA][Mn(dca)3].*

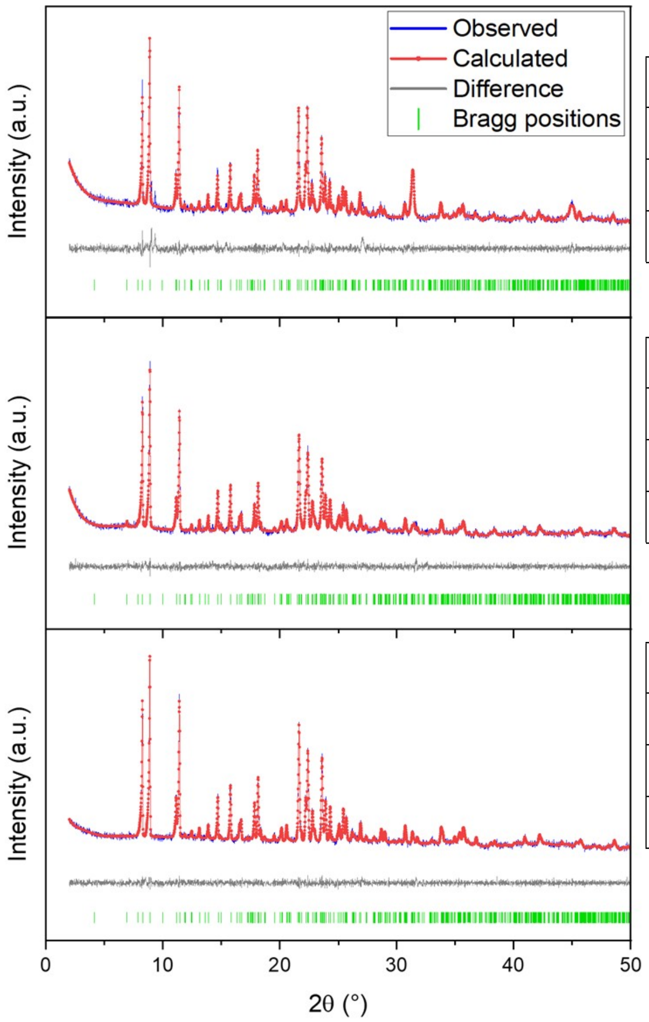

R_{wp}	Space Group	Calculated lattice parameters	Reported lattice parameters[7]
4.35	$P2_12_12$	a = 15.8609 (4) Å	a = 15.7971 (7) Å
		b = 15.8909 (4) Å	b = 15.8093 (9) Å
**Structure type**		c = 21.4068 (4) Å	c = 21.4138 (11) Å
		α = 90°	α = 90°
Triple-Rutile		β = 90°	β = 90°
		γ = 90°	γ = 90°

R_{wp}	Space Group	Calculated lattice parameters	Reported lattice parameters[7]
3.68	$P2_12_12$	a = 15.8361 (4) Å	a = 15.7971 (7) Å
		b = 15.8654 (4) Å	b = 15.8093 (9) Å
**Structure type**		c = 21.3791 (4) Å	c = 21.4138 (11) Å
		α = 90°	α = 90°
Triple-Rutile		β = 90°	β = 90°
		γ = 90°	γ = 90°

R_{wp}	Space Group	Calculated lattice parameters	Reported lattice parameters[7]
2.69	$P2_12_12$	a = 15.8312 (4) Å	a = 15.7971 (7) Å
		b = 15.8563 (4) Å	b = 15.8093 (9) Å
**Structure type**		c = 21.3693 (4) Å	c = 21.4138 (11) Å
		α = 90°	α = 90°
Triple-Rutile		β = 90°	β = 90°
		γ = 90°	γ = 90°

Fig. S4 *Pawley refinements for 3 repeats of the mechanochemically synthesised [TBuA][Fe(dca)3].*

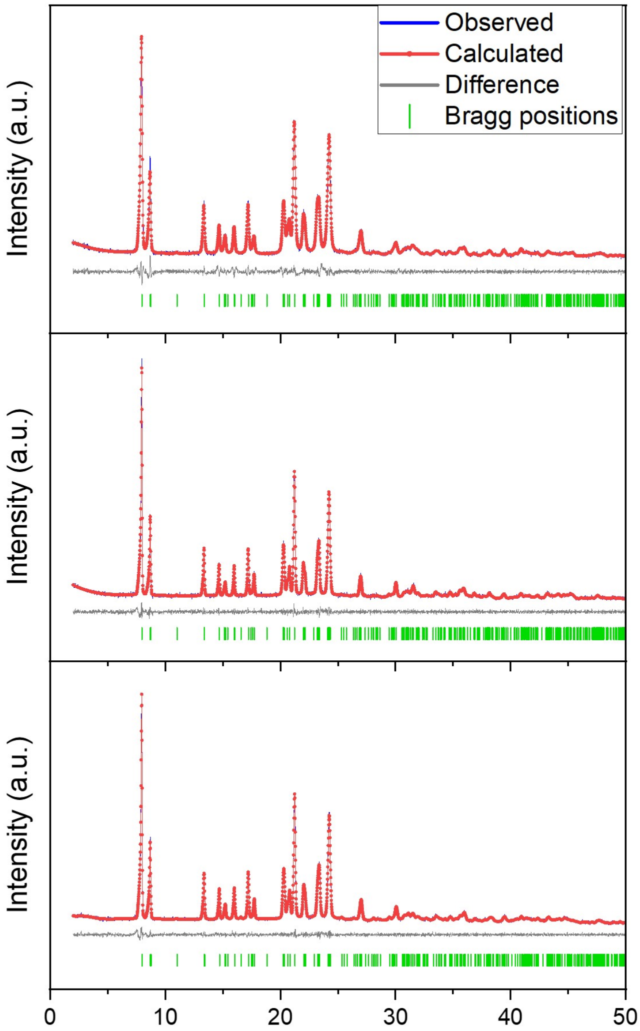

[5]R_{wp}	Space Group	Calculated lattice parameters	Reported lattice parameters[5]
4.63	Pnna	a = 13.2055 (4) Å	a = 13.2236 (6) Å
		b = 11.6199 (4) Å	b = 11.6300 (6) Å
**Structure type**		c = 20.2877 (4) Å	c = 20.3176 (9) Å
		α = 90°	α = 90°
$LiSbO_3$		β = 90°	β = 90°
		γ = 90°	γ = 90°

R_{wp}	Space Group	Calculated lattice parameters	Reported lattice parameters[5]
5.09	Pnna	a = 13.2071 (4) Å	a = 13.2236 (6) Å
		b = 11.6195 (4) Å	b = 11.6300 (6) Å
**Structure type**		c = 20.2929 (4) Å	c = 20.3176 (9) Å
		α = 90°	α = 90°
$LiSbO_3$		β = 90°	β = 90°
		γ = 90°	γ = 90°

R_{wp}	Space Group	Calculated lattice parameters	Reported lattice parameters[5]
4.09	Pnna	a = 13.1839 (4) Å	a = 13.2236 (6) Å
		b = 11.6050 (5) Å	b = 11.6300 (6) Å
**Structure type**		c = 20.2610 (4) Å	c = 20.3176 (9) Å
		α = 90°	α = 90°
$LiSbO_3$		β = 90°	β = 90°
		γ = 90°	γ = 90°

Fig. S5 *Pawley refinements for 3 repeats of the mechanochemically synthesised [TPnA][Mn(dca)3].*

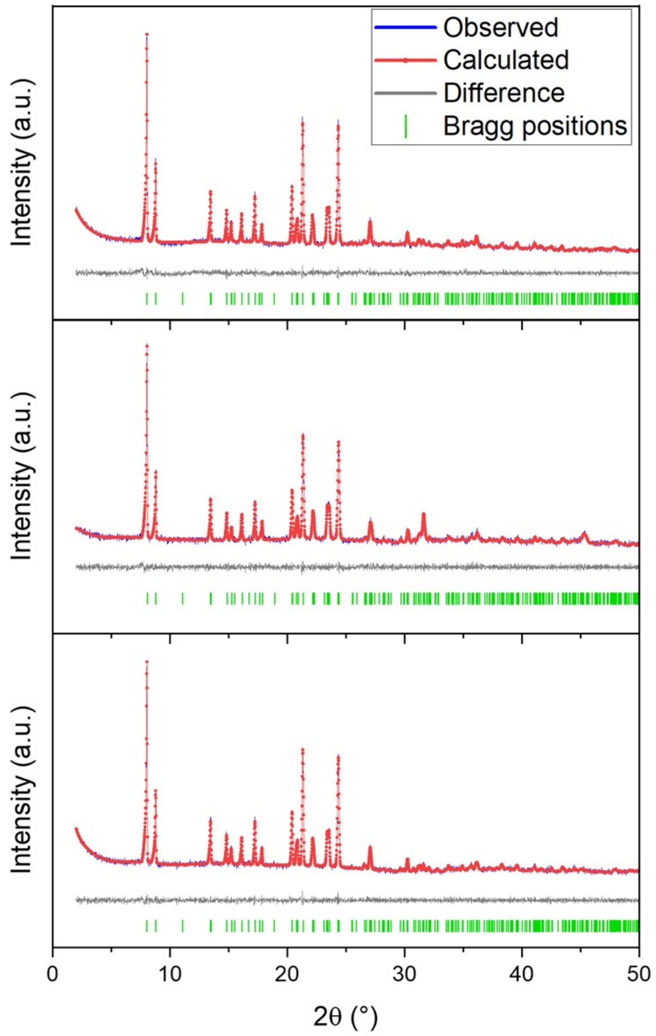

R_{wp}	Space Group	Calculated lattice parameters	Reported lattice parameters[6]
3.50	Pnna	a = 13.1215 (4) Å	a = 13.1007 (6) Å
		b = 11.6215 (4) Å	b = 11.6128 (5) Å
**Structure type**		c = 20.0894 (4) Å	c = 20.0929 (8) Å
		α = 90°	α = 90°
$LiSbO_3$		β = 90°	β = 90°
		γ = 90°	γ = 90°

R_{wp}	Space Group	Calculated lattice parameters	Reported lattice parameters[6]
4.78	Pnna	a = 13.0947 (4) Å	a = 13.1007 (6) Å
		b = 11.6051 (4) Å	b = 11.6128 (5) Å
**Structure type**		c = 20.0618 (4) Å	c = 20.0929 (8) Å
		α = 90°	α = 90°
$LiSbO_3$		β = 90°	β = 90°
		γ = 90°	γ = 90°

R_{wp}	Space Group	Calculated lattice parameters	Reported lattice parameters[6]
3.20	Pnna	a = 13.1078 (4) Å	a = 13.1007 (6) Å
		b = 11.6132 (5) Å	b = 11.6128 (5) Å
**Structure type**		c = 20.0849 (4) Å	c = 20.0929 (8) Å
		α = 90°	α = 90°
$LiSbO_3$		β = 90°	β = 90°
		γ = 90°	γ = 90°

Fig. S6 *Pawley refinements for 3 repeats of the mechanochemically synthesised [TPnA][Fe(dca)3].*

Thermogravimetric analysis (TGA)

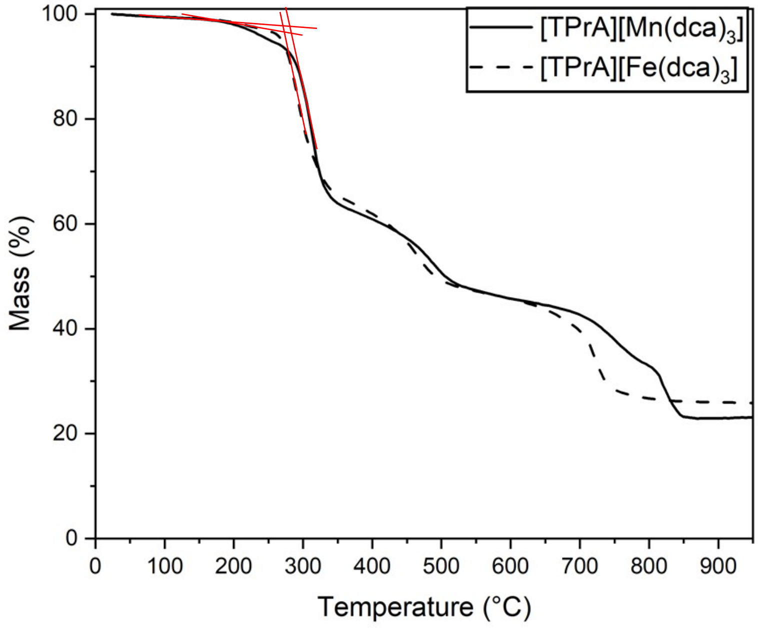

Fig. S7 *Thermogravimetric analysis of [TPrA][M(dca)$_3$] (M=Mn^{2+} (solid line) or Fe^{2+}(dashed line)) collected from 25 – 950 °C at a heating rate of 10 °C min^{-1} under an N$_2$ atmosphere.* T_d onset is indicated by the intersection of the respective red lines.

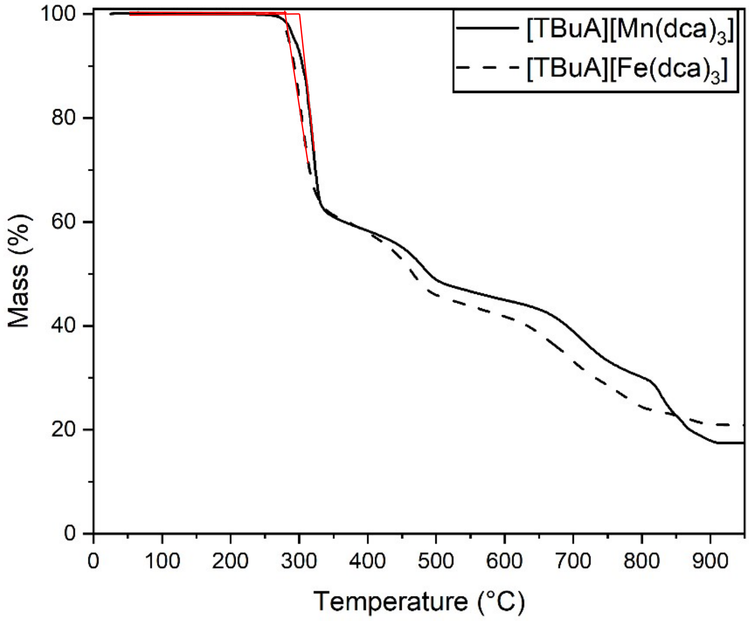

Fig. S8 *Thermogravimetric analysis of [TBuA][M(dca)$_3$] (M=Mn^{2+} (solid line) or Fe^{2+}(dashed line)) collected from 25 – 950 °C at a heating rate of 10 °C min^{-1} under an N$_2$ atmosphere.* T_d onset is indicated by the intersection of the respective red lines.

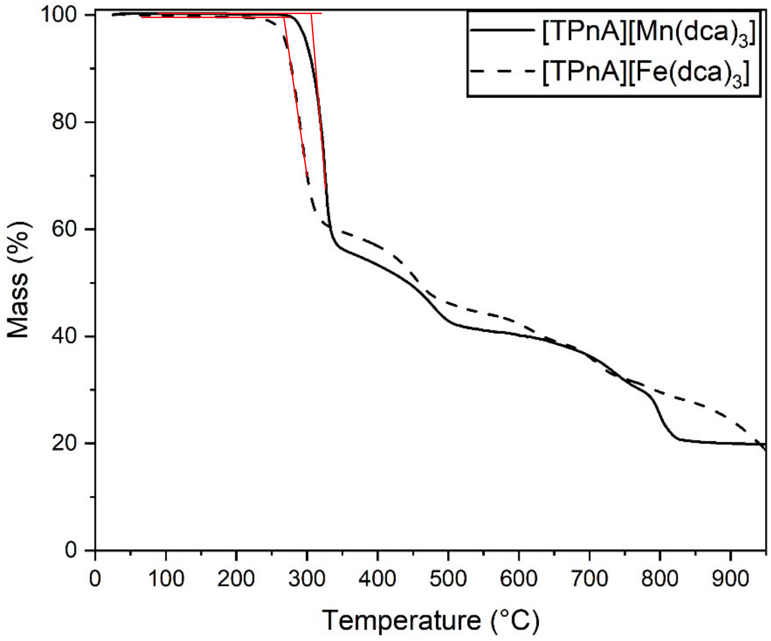

Fig. S9 *Thermogravimetric analysis of [TPnA][M(dca)$_3$] (M=Mn^{2+} (solid line) or Fe^{2+}(dashed line)) collected from 25 – 950 °C at a heating rate of 10 °C min^{-1} under an N_2 atmosphere.* T_d onset is indicated by the intersection of the respective red lines.

Differential scanning calorimetry

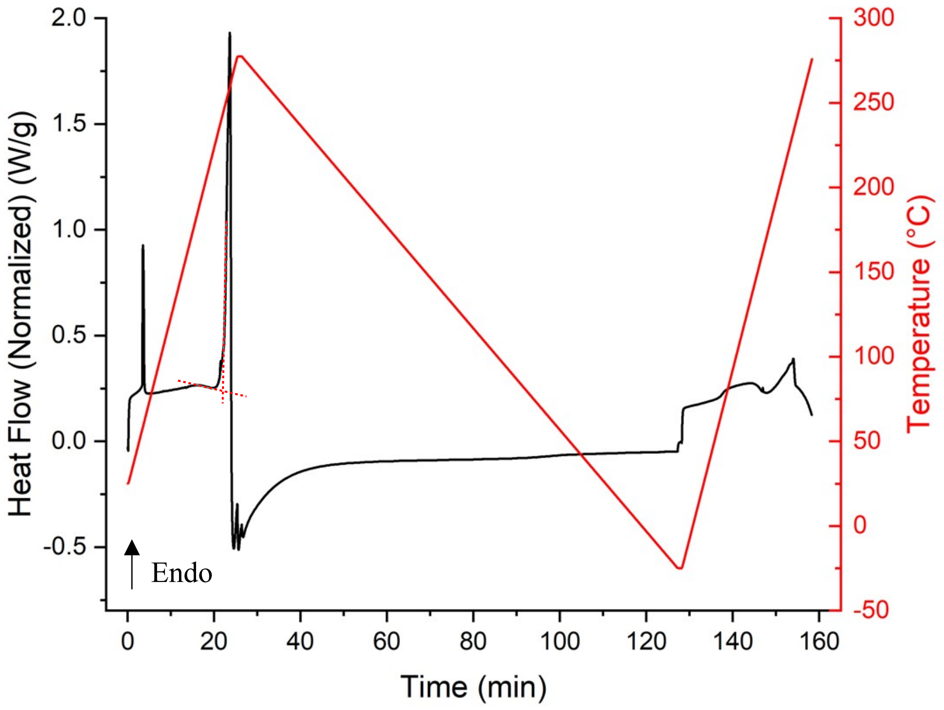

Fig. S10 *DSC data for [TPrA][Mn(dca)$_3$] with Heat flow (black trace) and temperature (red trace) plotted against time. Heat flow has been normalised to sample mass.* T_m onset is indicated the intersection of the dotted red lines. *The endothermic event at ca. 60 °C on the first up scan is a polymorphic transition.*

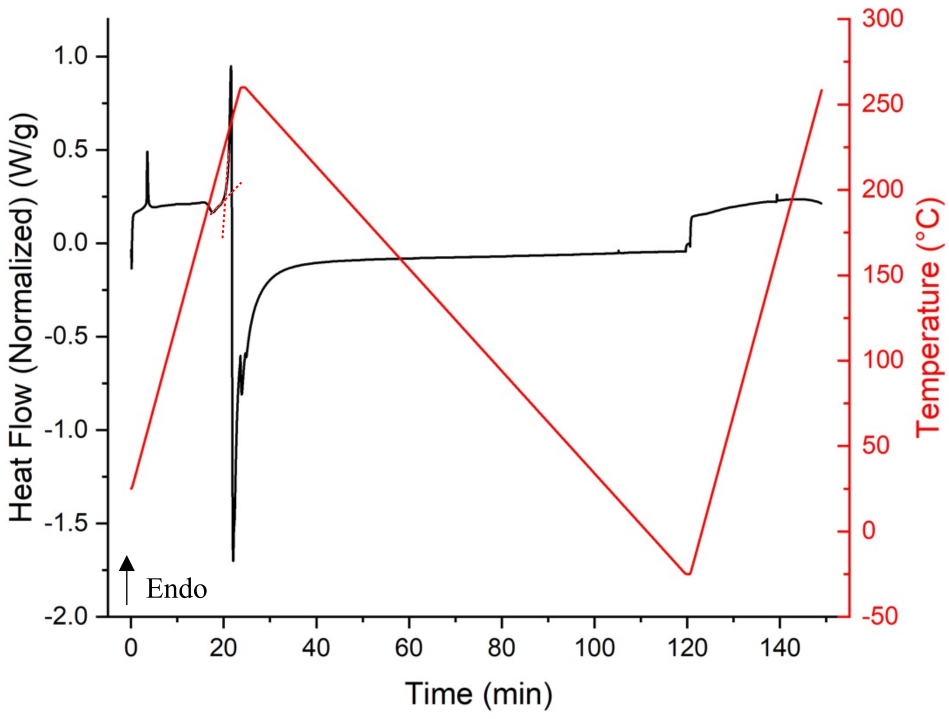

Fig. S11 *DSC data for [TPrA][Fe(dca)$_3$] with Heat flow (black trace) and temperature (red trace) plotted against time. Heat flow has been normalised to sample mass.* T_m onset is indicated the intersection of the dotted red lines. *The endothermic event at ca. 60 °C on the first up scan is a polymorphic transition.*

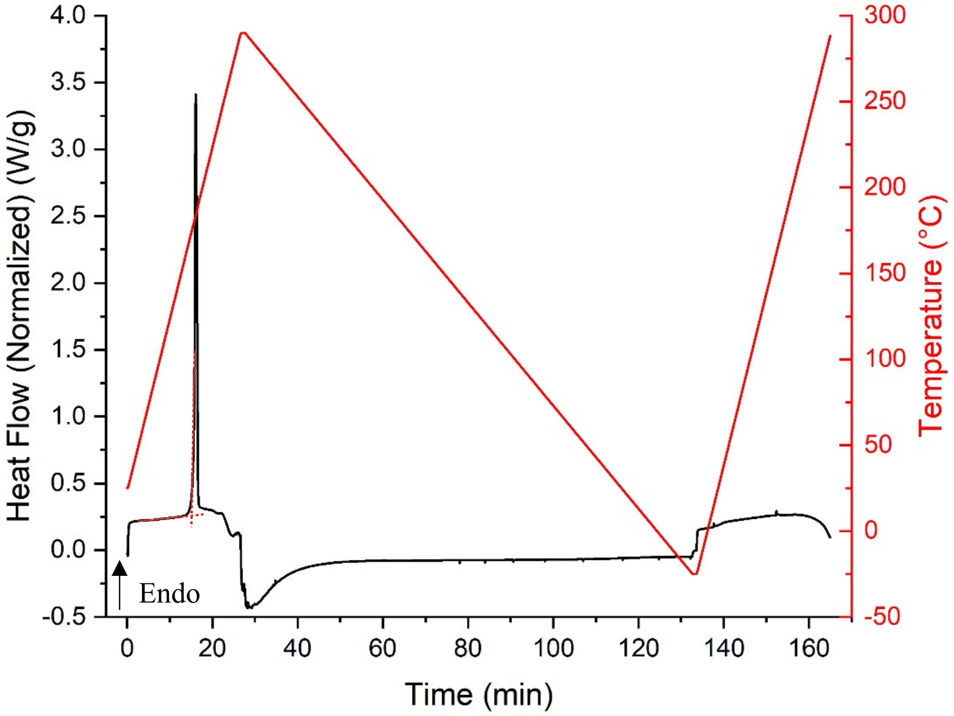

***Fig. S12** DSC data for [TBuA][Mn(dca)$_3$] with Heat flow (black trace) and temperature (red trace) plotted against time. Heat flow has been normalised to sample mass.* T_m onset is indicated the intersection of the dotted red lines.

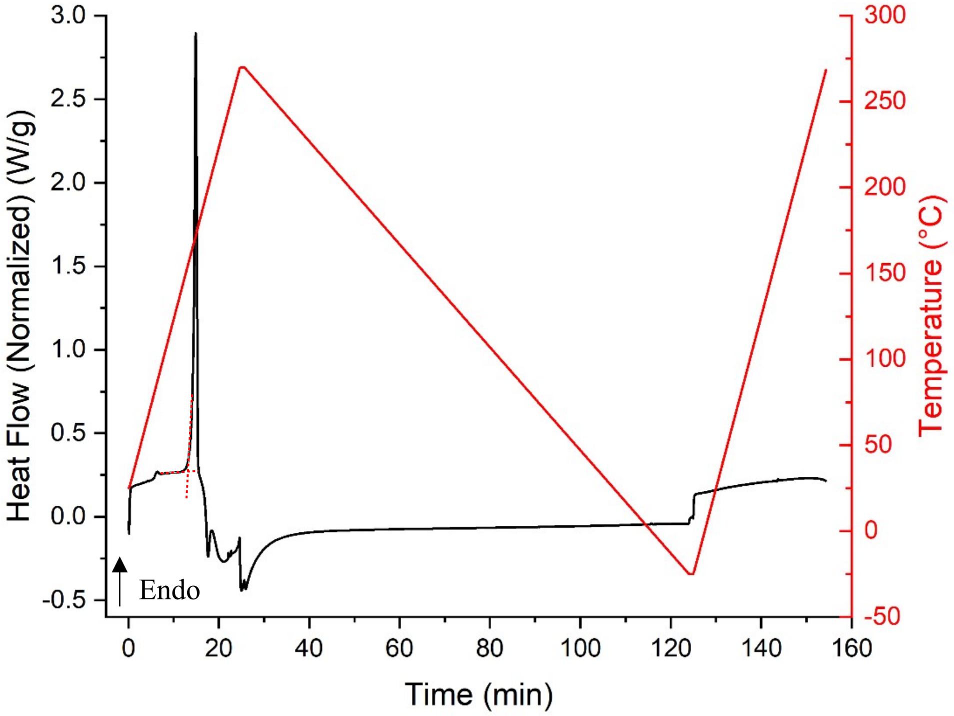

***Fig. S13** DSC data for [TBuA][Fe(dca)$_3$] with Heat flow (black trace) and temperature (red trace) plotted against time. Heat flow has been normalised to sample mass.* T_m onset is indicated the intersection of the dotted red lines.

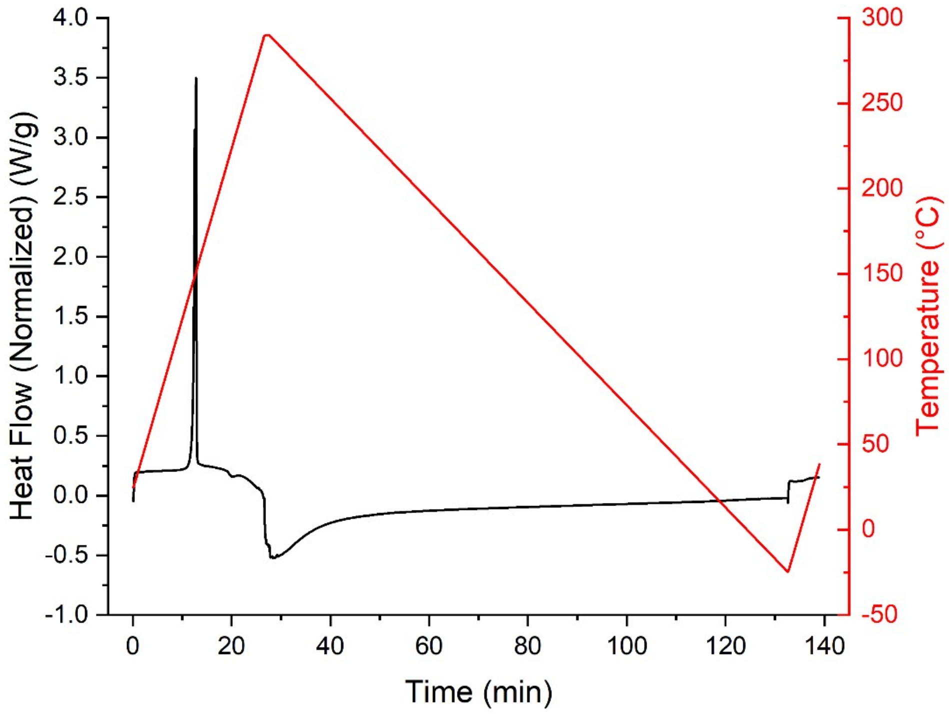

Fig. S14 *DSC data for [TPnA][Mn(dca)$_3$] with Heat flow (black trace) and temperature (red trace) plotted against time. Heat flow has been normalised to sample mass.* T_m onset is indicated the intersection of the dotted red lines.

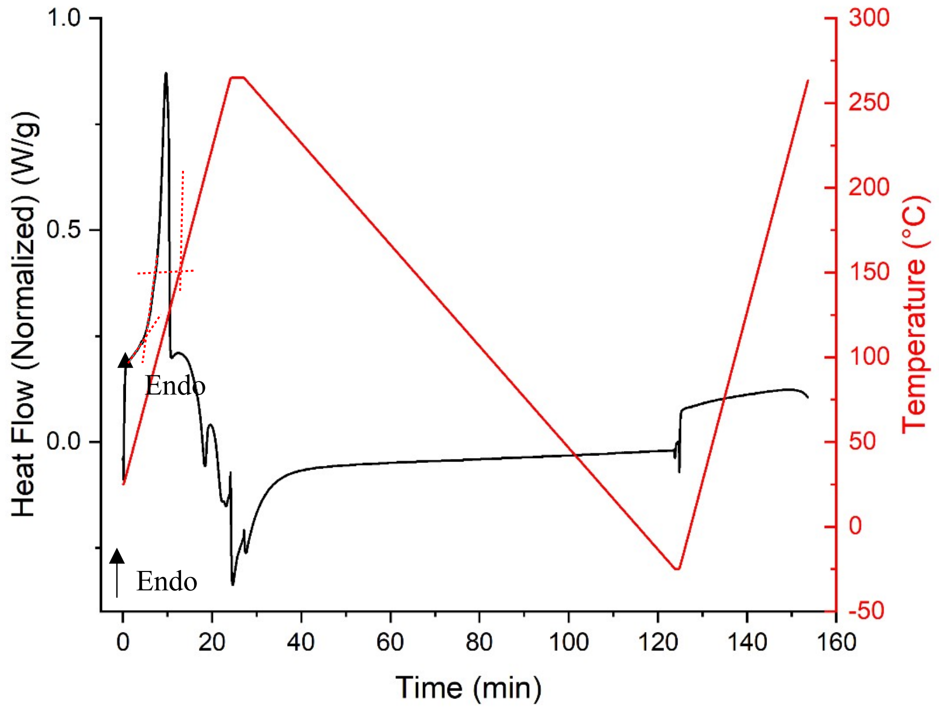

Fig. S15 *DSC data for [TPnA][Fe(dca)$_3$] with Heat flow (black trace) and temperature (red trace) plotted against time. Heat flow has been normalised to sample mass.* T_m onset is indicated the intersection of the dotted red lines.

CO_2 adsorption and desorption isotherms

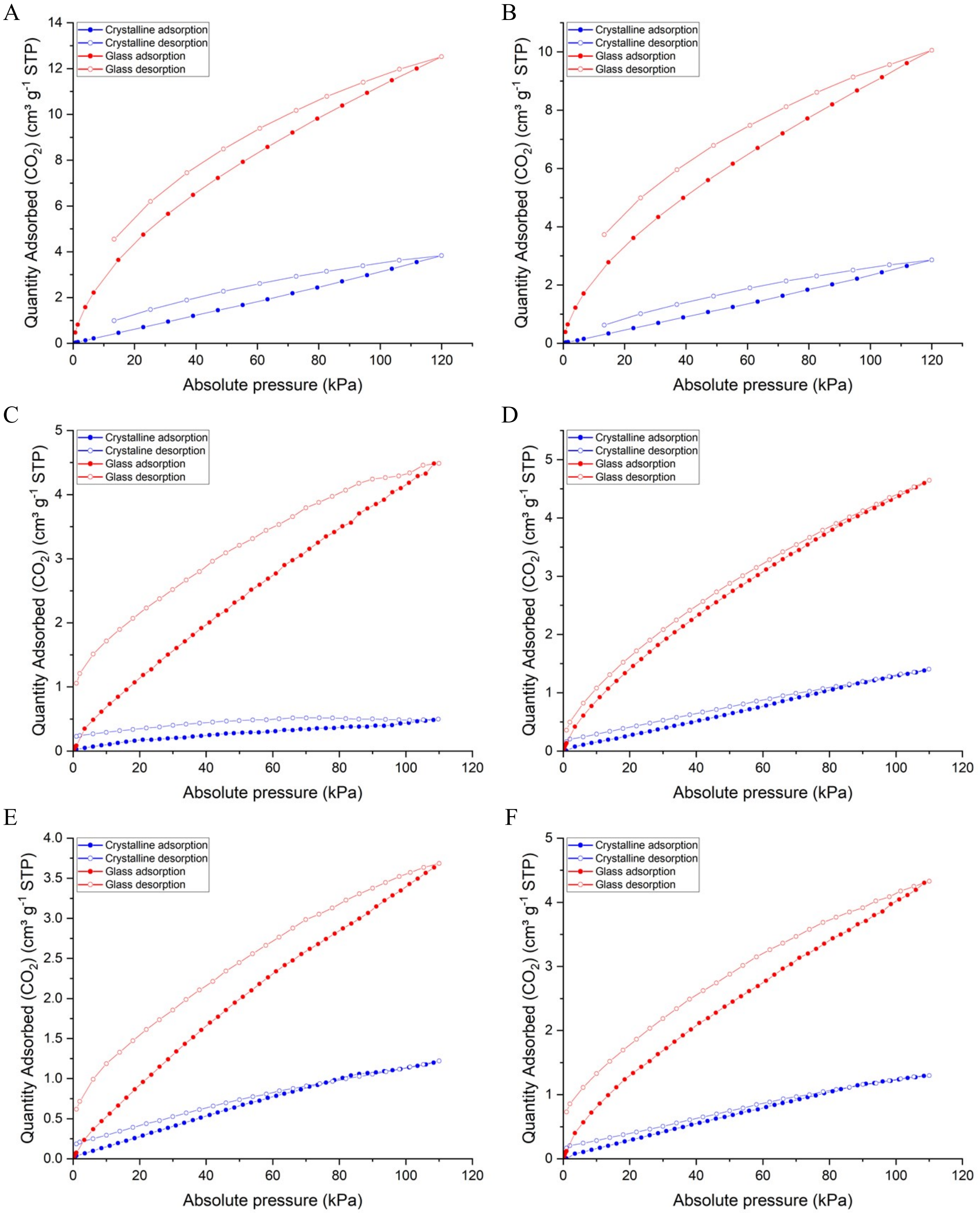

Fig, S16 *CO_2 adsorption and desorption isotherms collected at 0 °C for; a)* ***[TPrA][Mn(dca)$_3$]*** *(Blue) and a_g-****[TPrA][Mn(dca)$_3$]*** *(Red); b)* ***[TPrA][Fe(dca)3]*** *(Blue) and a_g-****[TPrA][Fe(dca)$_3$]*** *(Red); c)* ***[TBuA][Mn(dca)3]*** *(Blue) and a_g-****[TBuA][Mn(dca)$_3$]*** *(Red); d)* ***[TBuA][Fe(dca)3]*** *(Blue) and a_g-****[TBuA][Fe(dca)$_3$]*** *(Red); e)* ***[TPnA][Mn(dca)3]*** *(Blue) and a_g-****[TPnA][Mn(dca)$_3$]*** *(Red); f)* ***[TPnA][Fe(dca)3]*** *(Blue) and a_g-****[TPnA][Fe(dca)$_3$]*** *(Red).*

Scanning Electron Microscopy

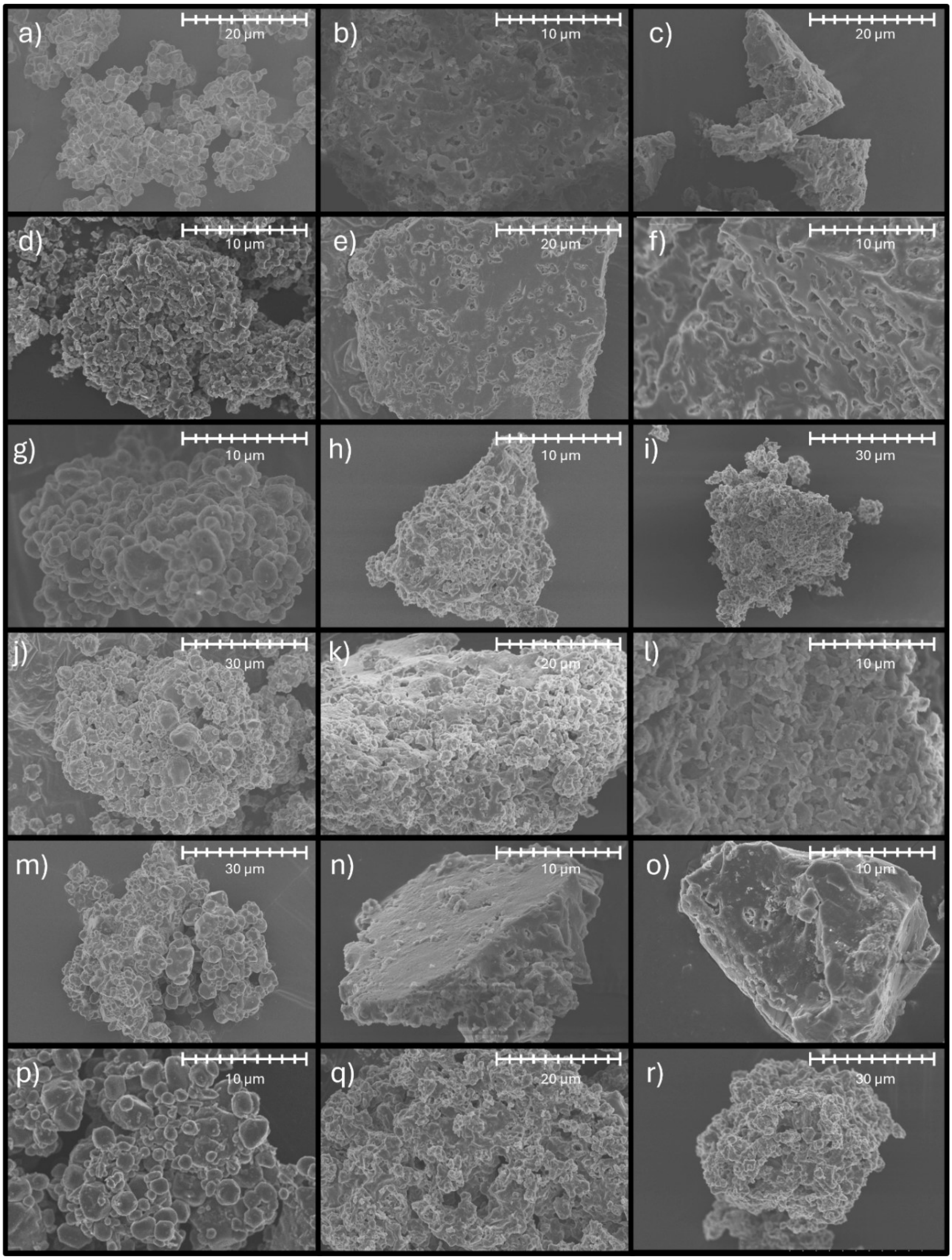

Fig. S17 *SEM images for; a)* ***[TPrA][Mn(dca)$_3$]****, b)* ***a_g-[TPrA][Mn(dca)$_3$]****, c) post water vapour exposure* ***a_g-[TPrA][Mn(dca)$_3$]****, d)* ***[TPrA][Fe(dca)$_3$]****, e)* ***a_g-[TPrA][Fe(dca)$_3$]****, f) post water vapour exposure* ***a_g-[TPrA][Fe(dca)$_3$]****,* g) ***[TBuA][Mn(dca)$_3$]****, h)* ***a_g-[TBuA][Mn(dca)$_3$]****, i) post water vapour exposure* ***a_g-[TBuA][Mn(dca)$_3$]****, j)* ***[TBuA][Fe(dca)$_3$]****, k)* ***a_g-[TBuA][Fe(dca)$_3$]****, l) post water vapour exposure* ***a_g-[TBuA][Fe(dca)$_3$]****, m)* ***[TPnA][Mn(dca)$_3$]****, n)* ***a_g-[TPnA][Mn(dca)$_3$]****, o) post water vapour exposure* ***a_g-[TPnA][Mn(dca)$_3$]****, p)* ***[TPnA][Fe(dca)$_3$]****, q)* ***a_g-[TPnA][Fe(dca)$_3$]*** *and r) post water vapour exposure* ***a_g-[TPnA][Fe(dca)$_3$]****.*

Water vapour stability

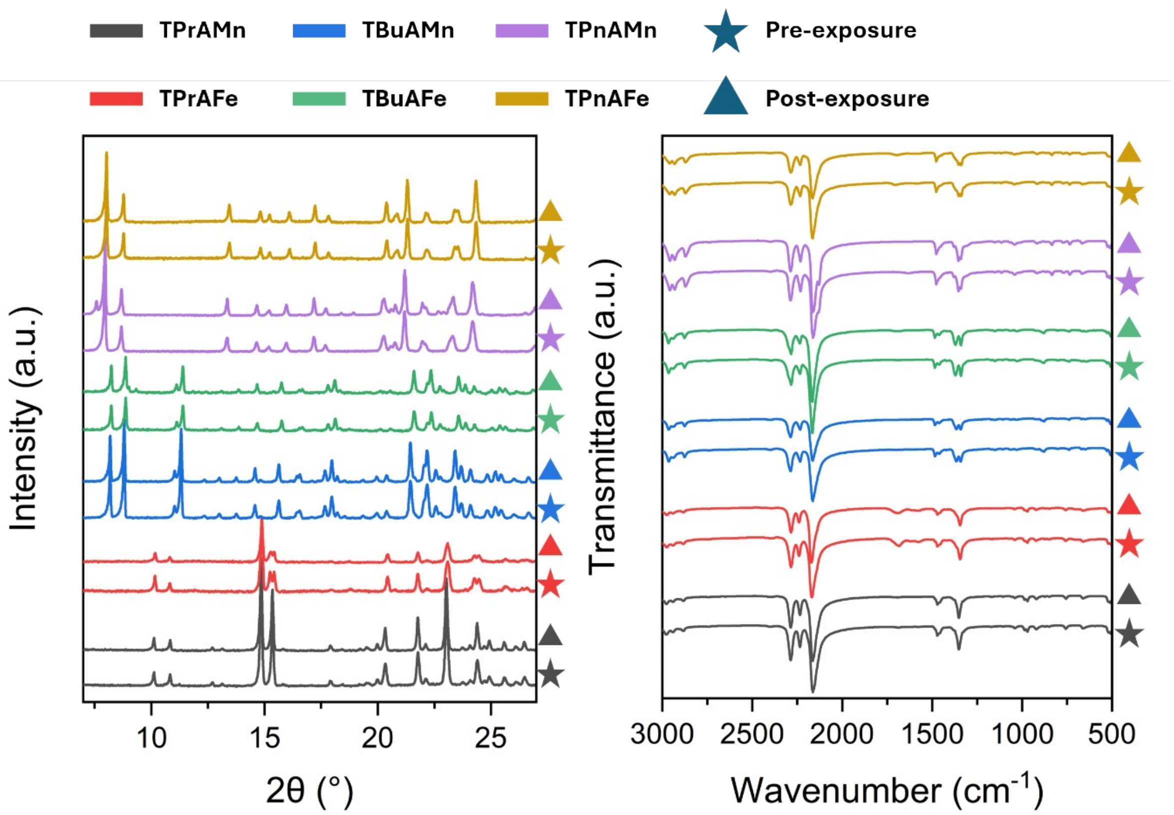

Fig. S18 *An enlarged version of Figure 3 from the main text. PXRD (left) and FTIR (right) analysis of Pre water vapour exposure (Pre-WV)(Star) and Post water vapour exposure (Post-WV)(Triangle) for **TPrAMn** (grey), **TPrAFe** (red), **TBuAMn** (blue), **TBuAFe** (green), **TPnAMn** (purple) and **TPnAFe** (gold).*

Water sorption isotherms

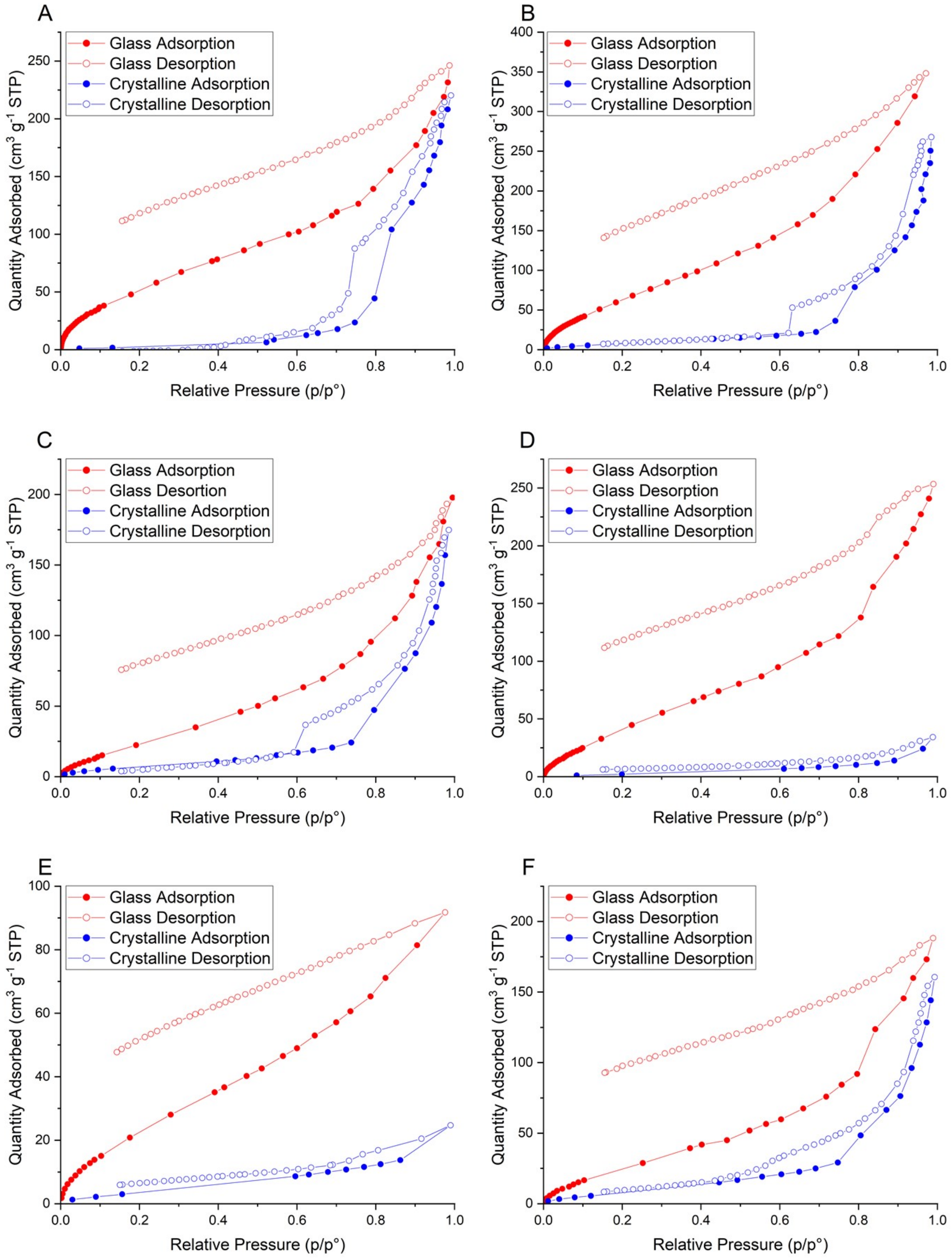

Fig. S19 *Water adsorption and desorption isotherms collected at 20 °C for; a)* ***[TPrA][Mn(dca)$_3$]*** *(Blue) and a$_g$-****[TPrA][Mn(dca)$_3$]*** *(Red); b)* ***[TPrA][Fe(dca)$_3$]*** *(Blue) and a$_g$-****[TPrA][Fe(dca)$_3$]*** *(Red); c)* ***[TBuA][Mn(dca)$_3$]*** *(Blue) and a$_g$-****[TBuA][Mn(dca)$_3$]*** *(Red); d)* ***[TBuA][Fe(dca)$_3$]*** *(Blue) and a$_g$-****[TBuA][Fe(dca)$_3$]*** *(Red); e)* ***[TPnA][Mn(dca)$_3$]*** *(Blue) and a$_g$-****[TPnA][Mn(dca)$_3$]*** *(Red); f)* ***[TPnA][Fe(dca)3]*** *(Blue) and a$_g$-****[TPnA][Fe(dca)$_3$]*** *(Red).*

Water contact angle measurements

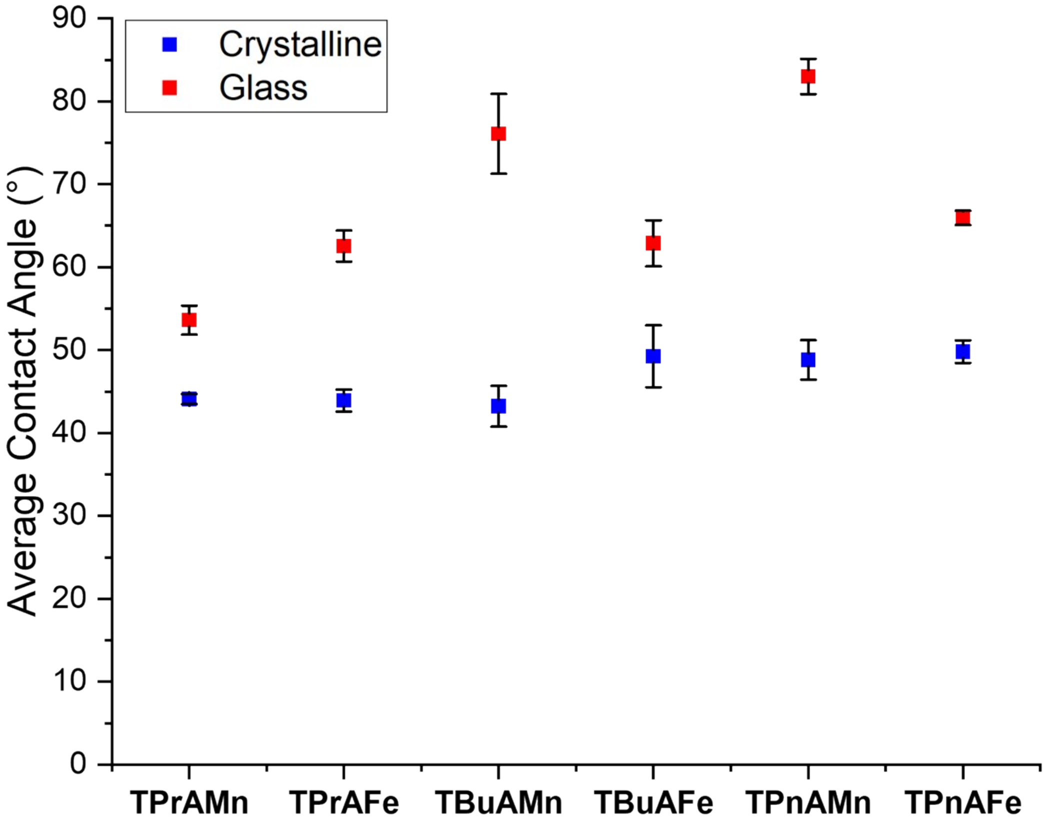

Fig. S20 *Average water contact angle and standard deviation for all crystalline (blue) and glass (red) HOIPs.*

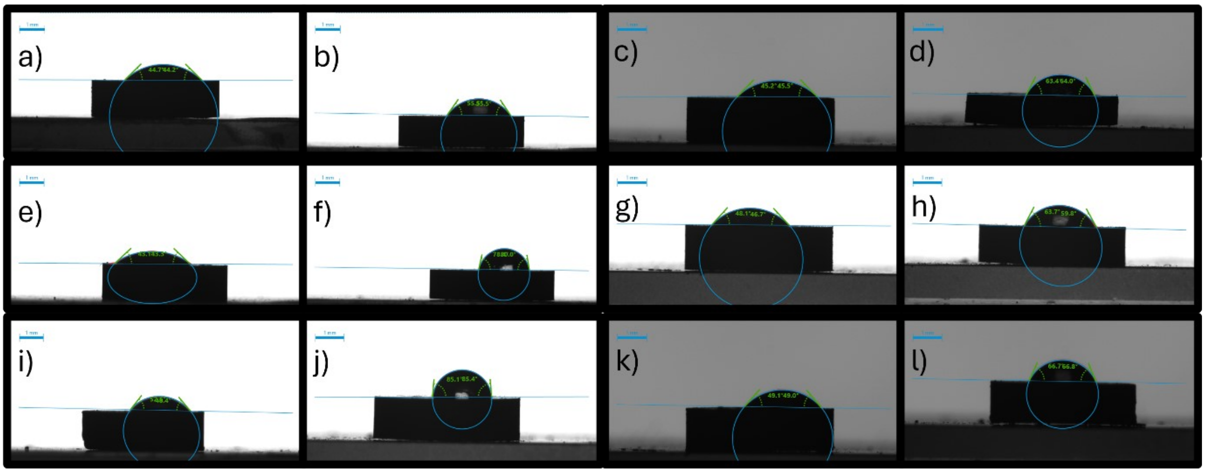

Fig. S21 *Select examples of measured water drops for; a)* ***[TPrA][Mn(dca)$_3$]****, b)* ***a_g-[TPrA][Mn(dca)$_3$]****, c)* ***[TPrA][Fe(dca)$_3$]****, d)* ***a_g-[TPrA][Fe(dca)$_3$]****, e)* ***[TBuA][Mn(dca)$_3$]****, f)* ***a_g-[TBuA][Mn(dca)$_3$]****, g)* ***[TBuA][Fe(dca)$_3$]****, h)* ***a_g-[TBuA][Fe(dca)$_3$]****, i)* ***[TPnA][Mn(dca)$_3$]****, j)* ***a_g-[TPnA][Mn(dca)$_3$]****, k)* ***[TPnA][Fe(dca)$_3$]****, l)* ***a_g-[TPnA][Fe(dca)$_3$]****.*

FTIR

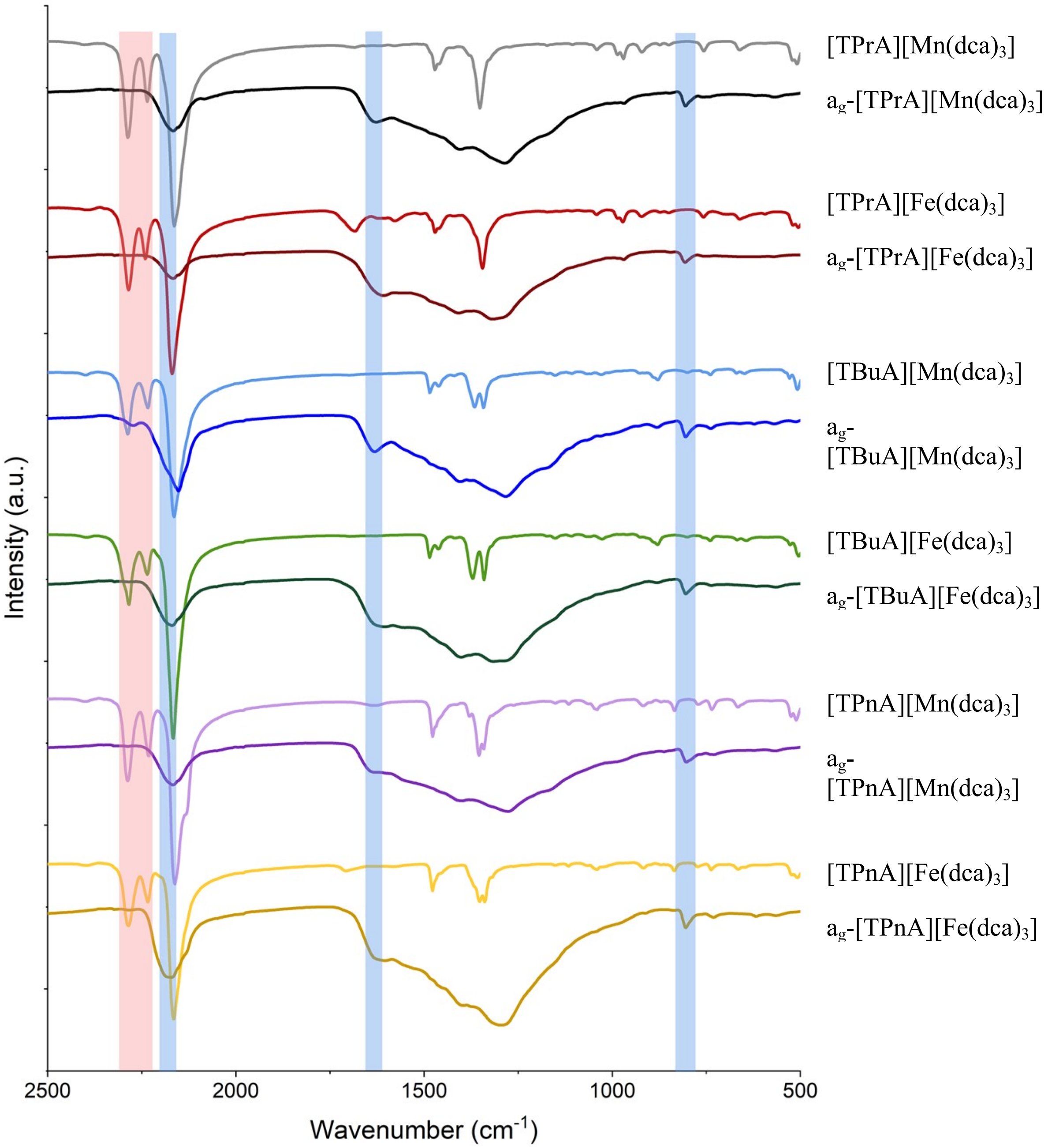

Fig. S22 *FTIR spectra of the crystalline and glassy states of all 6 HOIP systems. The red band highlights the loss of peaks at ~2285 and 2230 cm^{-1}, corresponding to v_sC≡N and v_{as}C-N + v_sC-N of the dicyanamide respectively. The loss of these peaks upon glass formation suggest that the overall concentration of nitrile groups is significantly reduced, this is attributed to the formation of tricyanomelaminate units. The left-most blue band ~2175 cm^{-1} corresponds to v_{as}C≡N still present in the system at significantly high enough quantities to be clearly detected. The central blue band corresponds to 1653 cm^{-1} v_sRing N, characteristic of the tricyanomelaminate unit. The right-most blue band highlights the 804 cm^{-1} δ_{as}N-C-N, another characteristic peak for the trimer product.[8] With the loss of characteristics peaks for dicyanamide and the emergence of peaks for the trimerized species, we conclude that a sizeable portion of the dicyanamide species has ultimately formed the trimer upon melt-quenching.*

UV-Vis reflectance data

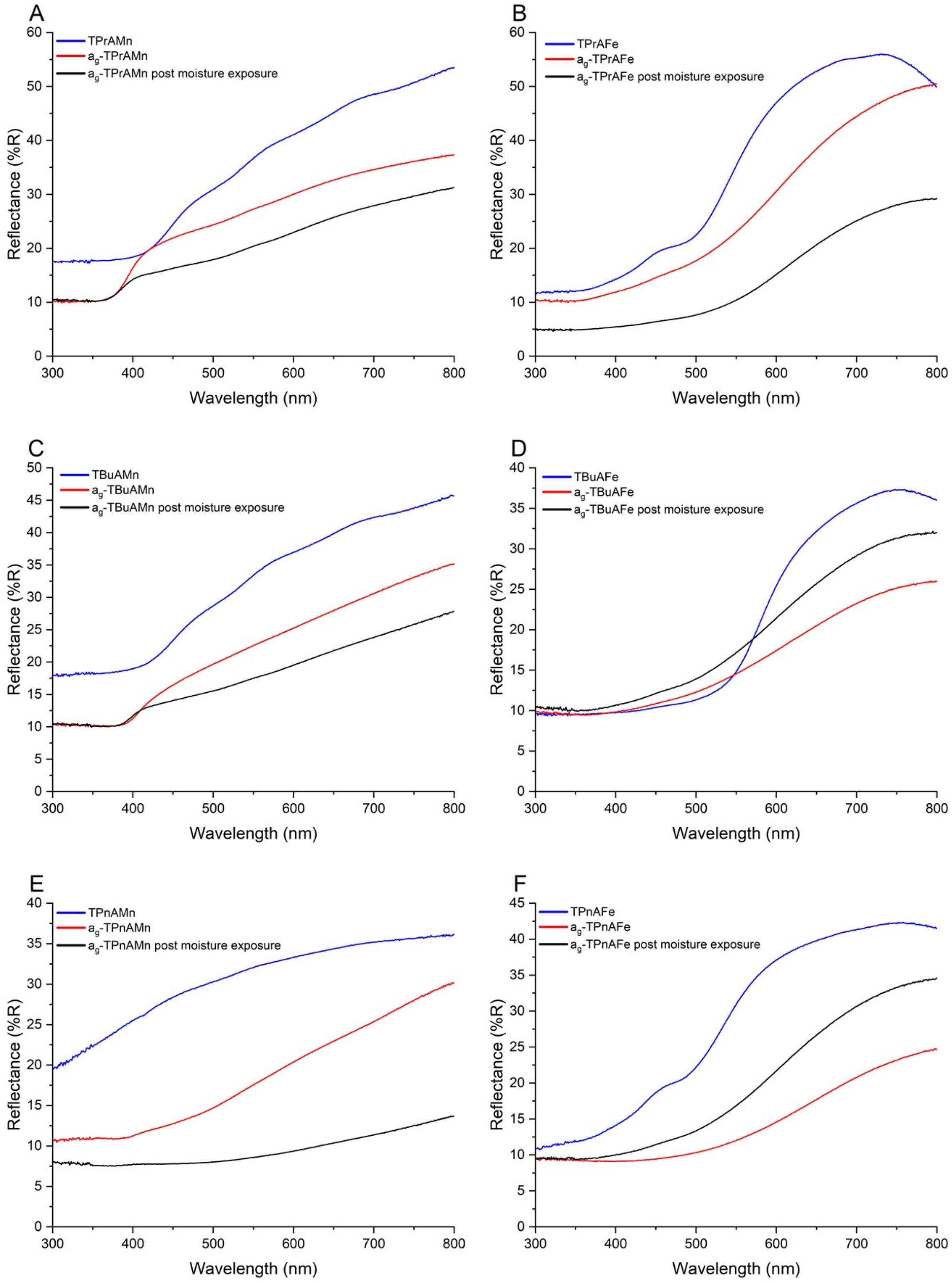

Fig. S23 *UV-Vis reflectance data from 300 - 800 nm for; a)* ***[TPrA][Mn(dca)$_3$]*** *(Blue),* ***a_g-[TPrA][Mn(dca)$_3$]*** *(Red) and post water exposure* ***a_g-[TPrA][Mn(dca)$_3$]*** *(Black); b)* ***[TPrA][Fe(dca)$_3$]*** *(Blue),* ***a_g-[TPrA][Fe(dca)$_3$]*** *(Red) and post water exposure* ***a_g-[TPrA][Mn(dca)$_3$]*** *(Black); c)* ***[TBuA][Mn(dca)$_3$]*** *(Blue),* ***a_g-[TBuA][Mn(dca)$_3$]*** *(Red) and post water exposure* ***a_g-[TBuA][Mn(dca)$_3$]*** *(Black); d)* ***[TBuA][Fe(dca)$_3$]*** *(Blue),* ***a_g-[TBuA][Fe(dca)$_3$]*** *(Red) and post water exposure* ***a_g-[TBuA][Fe(dca)$_3$]*** *(Black); e)* ***[TPnA][Mn(dca)$_3$]*** *(Blue),* ***a_g-[TPnA][Mn(dca)$_3$]*** *(Red) and post water exposure* ***a_g-[TPnA][Mn(dca)$_3$]*** *(Black); d)* ***[TPnA][Fe(dca)$_3$]*** *(Blue),* ***a_g-[TPnA][Fe(dca)$_3$]*** *(Red) and post water exposure* ***a_g-[TPnA][Fe(dca)$_3$]*** *(Black).*

<u>Band gap calculations using the Kubelka-Munk function</u>

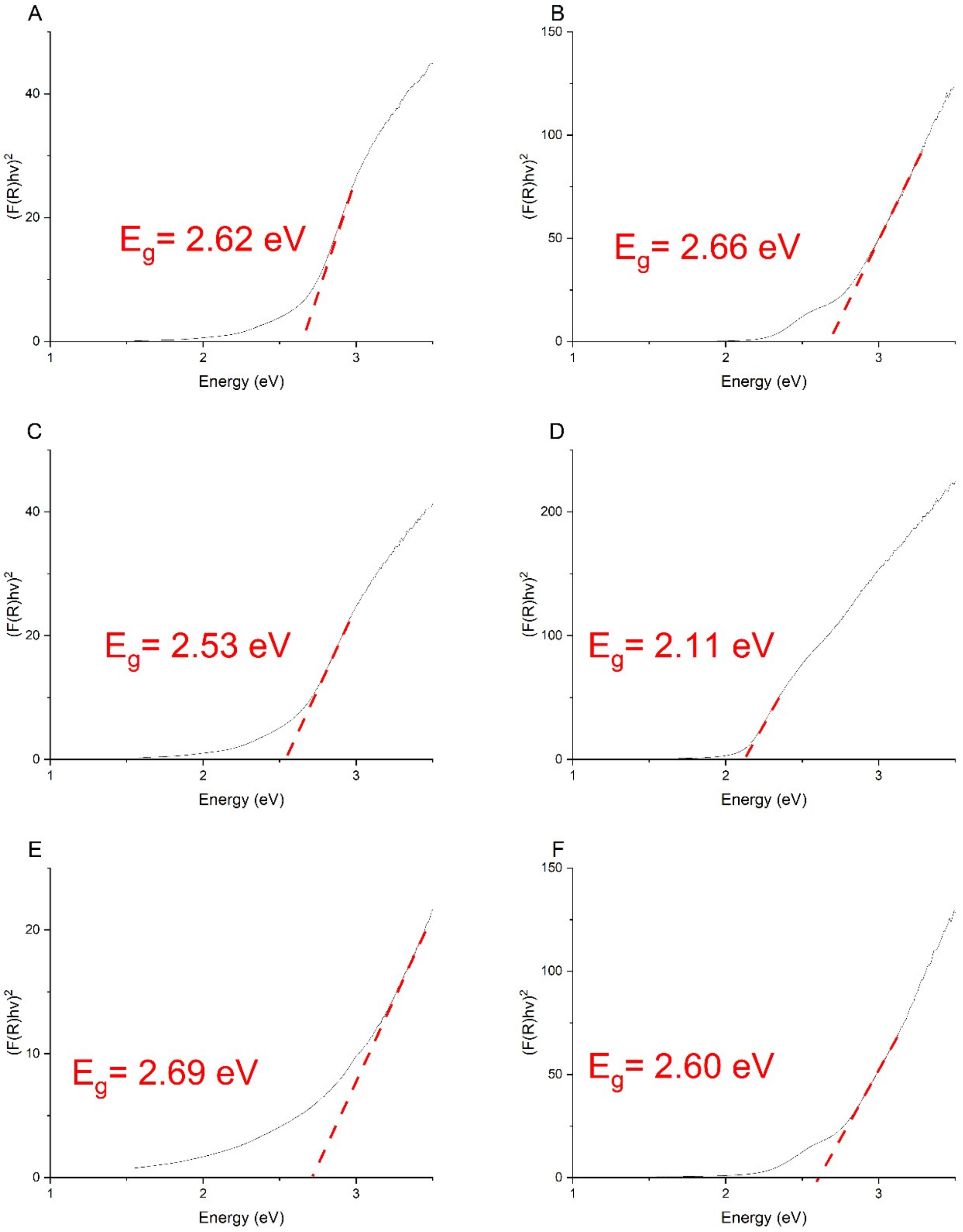

Fig. S24 *Plots of (F(R)hv)^2 vs energy as calculated from UV-Vis reflectance data for; a)* ***[TPrA][Mn(dca)$_3$]****; b)* ***[TPrA][Fe(dca)$_3$]****; c)* ***[TBuA][Mn(dca)$_3$]****; d)* ***[TBuA][Fe(dca)$_3$]****; e)* ***[TPnA][Mn(dca)$_3$]****; f)* ***[TPnA][Fe(dca)$_3$]****. Red line segments highlight the first linear region selected for calculation of optical band-gap. Inset table shows the parameters of the linear fit used to calculate x-intercept.*

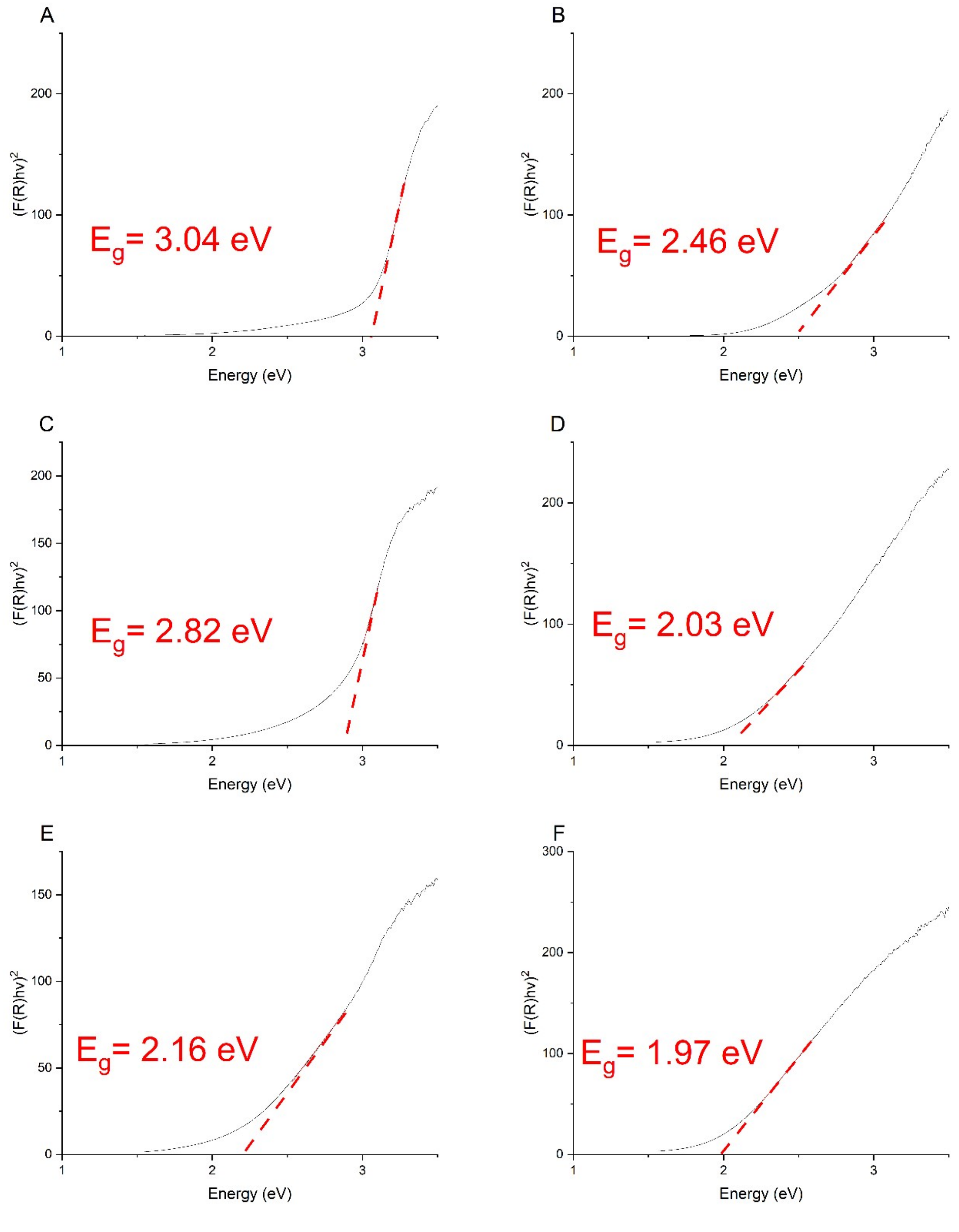

Fig. S25 *Plots of* $(F(R)hv)^2$ *vs energy as calculated from UV-Vis reflectance data for; a)* a_g-***[TPrA][Mn(dca)*** $_3$ ***]****; b)* a_g-***[TPrA][Fe(dca)*** $_3$ ***]****; c)* a_g-***[TBuA][Mn(dca)*** $_3$ ***]****; d)* a_g-***[TBuA][Fe(dca)*** $_3$ ***]****; e)* a_g-***[TPnA][Mn(dca)*** $_3$ ***]****; f)* a_g-***[TPnA][Fe(dca)*** $_3$ ***]****. Red line segments highlight the first linear region selected for calculation of optical band-gap. Inset table shows the parameters of the linear fit used to calculate x-intercept.*

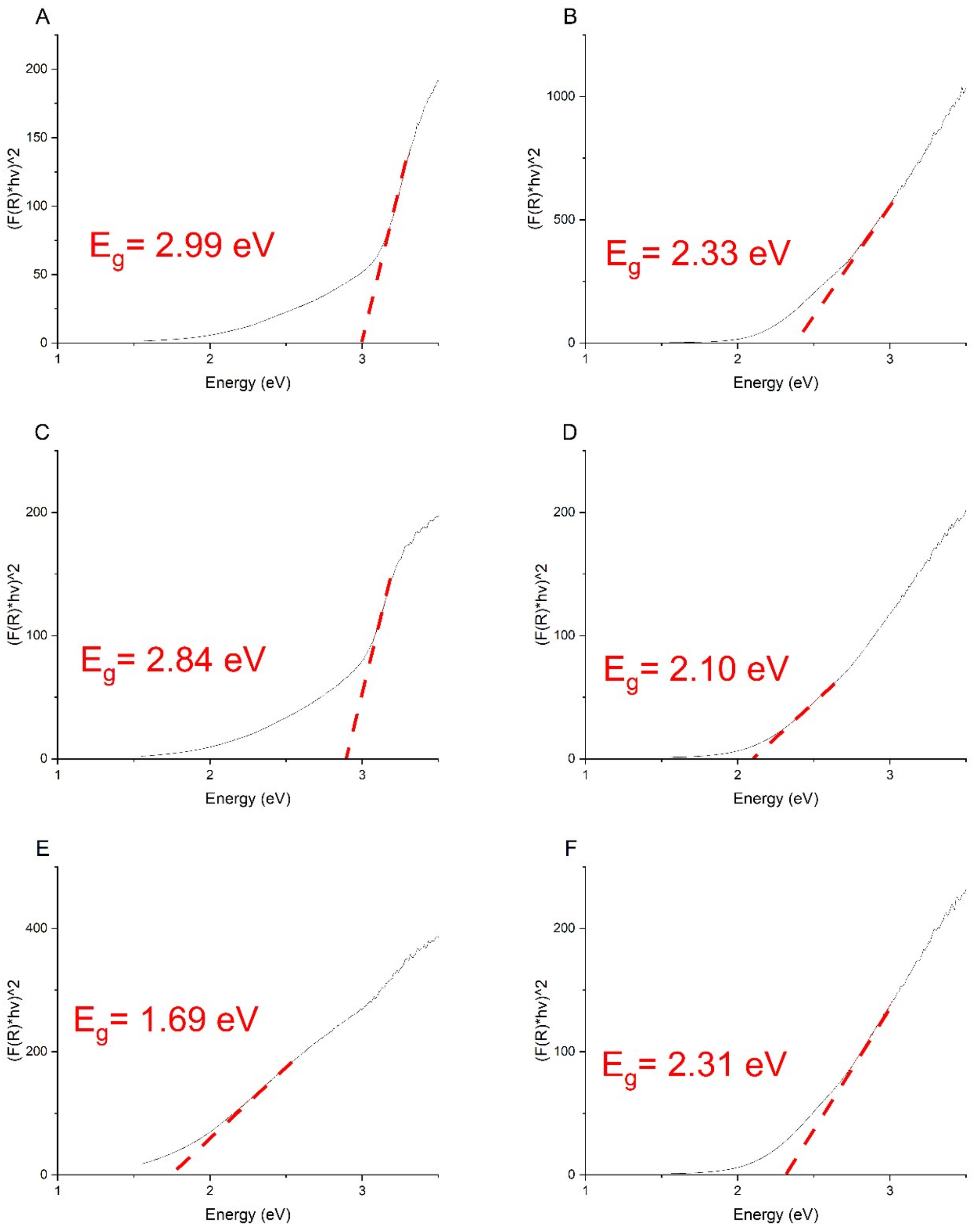

Fig. S26 *Plots of $(F(R)hv)^2$ vs energy as calculated from UV-Vis reflectance data for; a) H_2O saturated a_g-**[TPrA][Mn(dca)$_3$]**; b) H_2O saturated a_g-**[TPrA][Fe(dca)$_3$]**; c) H_2O saturated a_g-**[TBuA][Mn(dca)$_3$]**; d) H_2O saturated a_g-**[TBuA][Fe(dca)$_3$]**; e) H_2O saturated a_g-**[TPnA][Mn(dca)$_3$]**; f) H_2O saturated a_g-**[TPnA][Fe(dca)$_3$]**. Red line segments highlight the first linear region selected for calculation of optical band-gap. Inset table shows the parameters of the linear fit used to calculate x-intercept.*

<u>Photoluminescence spectroscopy – Emission Spectra</u>

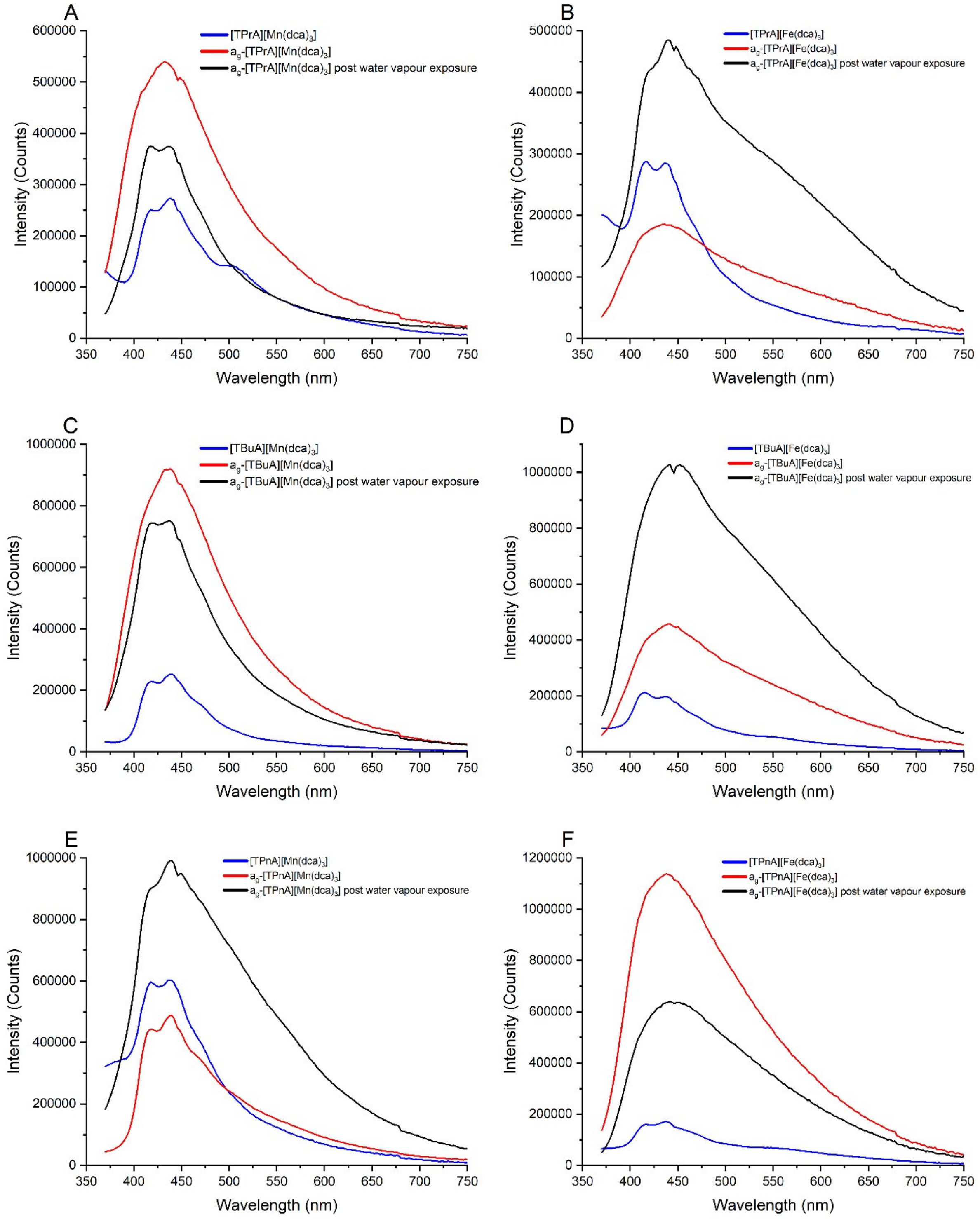

Fig. S27 *Photoluminescence emission spectra for; a)* ***[TPrA][Mn(dca)$_3$]*** *(Blue),* ***a$_g$-[TPrA][Mn(dca)$_3$]*** *(Red) and post water exposure* ***a$_g$-[TPrA][Mn(dca)$_3$]*** *(Black); b)* ***[TPrA][Fe(dca)$_3$]*** *(Blue),* ***a$_g$-[TPrA][Fe(dca)$_3$]*** *(Red) and post water exposure* ***a$_g$-[TPrA][Mn(dca)$_3$]*** *(Black); c)* ***[TBuA][Mn(dca)$_3$]*** *(Blue),* ***a$_g$-[TBuA][Mn(dca)$_3$]*** *(Red) and post water exposure* ***a$_g$-[TBuA][Mn(dca)$_3$]*** *(Black); d)* ***[TBuA][Fe(dca)$_3$]*** *(Blue),* ***a$_g$-[TBuA][Fe(dca)$_3$]*** *(Red) and post water exposure* ***a$_g$-[TBuA][Fe(dca)$_3$]*** *(Black); e)* ***[TPnA][Mn(dca)$_3$]*** *(Blue),* ***a$_g$-[TPnA][Mn(dca)$_3$]*** *(Red) and post water exposure* ***a$_g$-[TPnA][Mn(dca)$_3$]*** *(Black); f)* ***[TPnA][Fe(dca)$_3$]*** *(Blue),* ***a$_g$-[TPnA][Fe(dca)$_3$]*** *(Red) and post water exposure* ***a$_g$-[TPnA][Fe(dca)$_3$]*** *(Black).*

Conductivity measurements

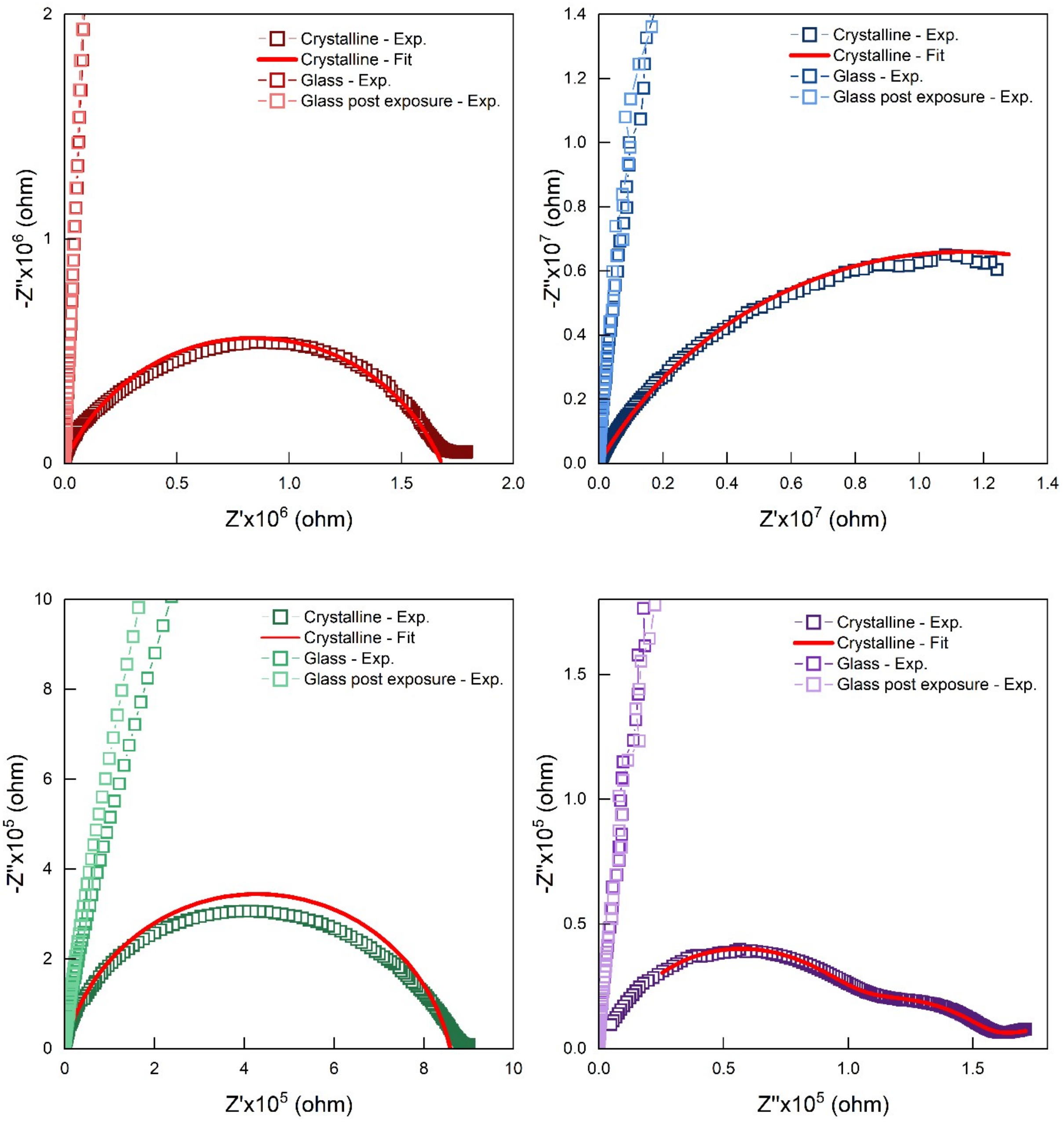

Fig. S28 *Nyquist plots of the crystalline, glass, and post water exposure glass states of [TPrA][Fe(dca)$_3$] (Red), [TBuA][Mn(dca)$_3$] (Blue), [TBuA][Fe(dca)$_3$] (Green) and [TPnA][Mn(dca)$_3$] (Purple) as measured by AC electrical impedance spectroscopy.*

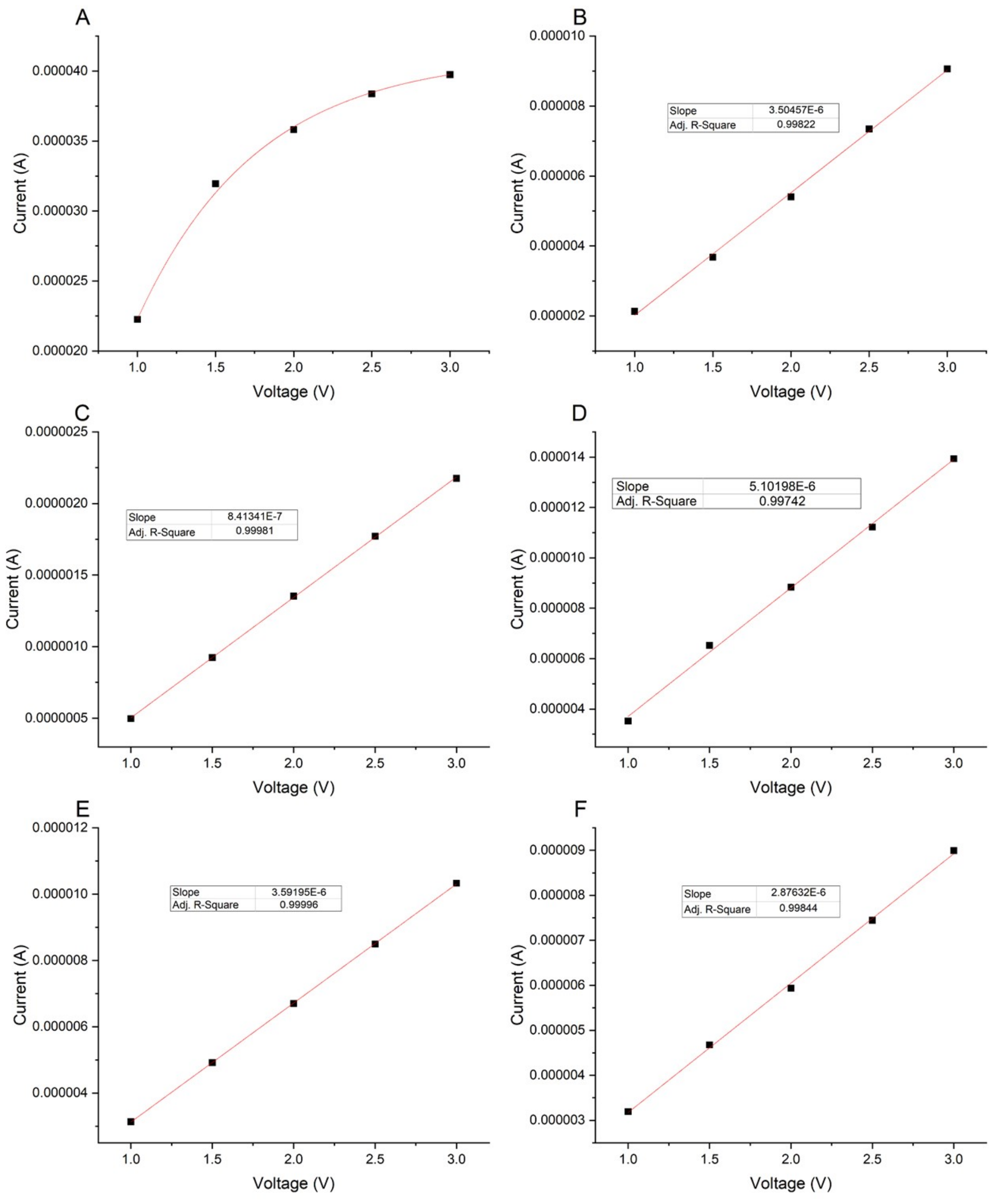

Fig. S29 *I-V measurements for a) [TPrA][Mn(dca)$_3$], b) [TPrA][Fe(dca)$_3$], c) [TBuA][Mn(dca)$_3$], d) [TBuA][Fe(dca)$_3$], e) [TPnA][Mn(dca)$_3$] and f) [TPnA][Fe(dca)$_3$].*

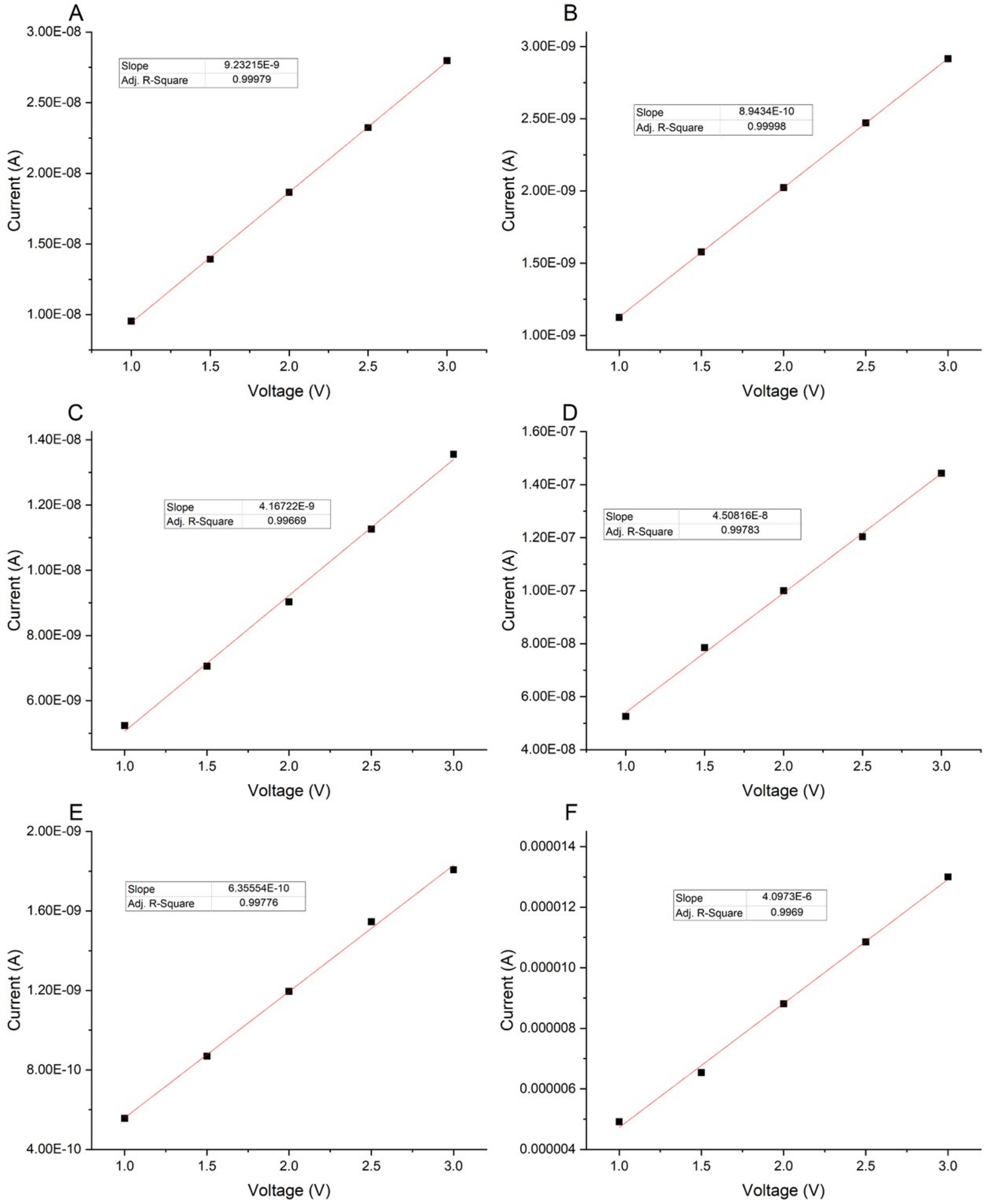

Fig. S30 *I-V measurements for a) a_g-[TPrA][Mn(dca)3], b) a_g-[TPrA][Fe(dca)3], c) a_g-[TBuA][Mn(dca)3], d) a_g-[TBuA][Fe(dca)3], e) a_g-[TPnA][Mn(dca)3] and f) a_g-[TPnA][Fe(dca)3].*

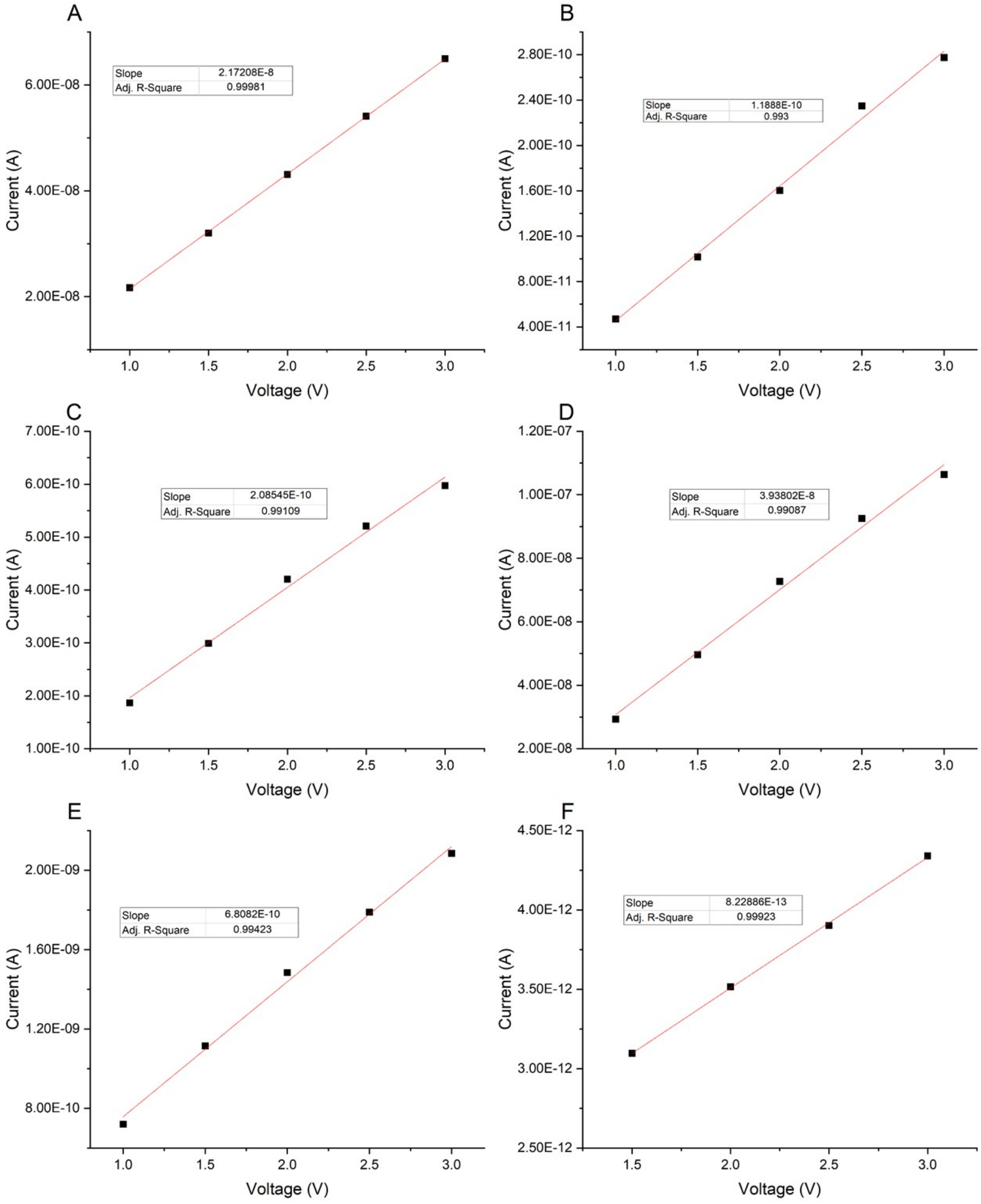

Fig. S31 *I-V measurements for the post water vapour exposure states of a) a_g-[TPrA][Mn(dca)3], b) a_g-[TPrA][Fe(dca)3], c) a_g-[TBuA][Mn(dca)3], d) a_g-[TBuA][Fe(dca)3], e) a_g-[TPnA][Mn(dca)3] and f) a_g-[TPnA][Fe(dca)3].*

Due to the high resistance at low frequencies, some AC EIS experimental data was unsuitable for fitting, therefore, only the relative trends can be extracted from Fig. S28.[9] It is clear that upon glass formation there is a significant increase in resistance, as demonstrated by the sharp increase in slope of the high frequency ranges shown in these plots. The data that could be confidently fit was done so using equivalent circuits and the calculated conductivity values are displayed below.

Measurements made using DC I-V methods allowed for all conductivities to be calculated with the exception of [TPrA][Mn(dca)$_3$] which was excluded due to non-ohmic behaviour.

Table S1 *Conductivity values for all HOIPs in the crystalline, glass and glass post exposure to water vapour, measured using AC electrical impedance spectroscopy and DC I-V measurements.*

HOIP	State	Average conductivity (S cm^{-1}) (AC EIS method)	EIS Error (%)	Conductivity (S cm^{-1}) (DC I-V method)
[TPrA][Mn(dca)$_3$]	Crystalline	1.9×10^{-4}	3.36	(Non-Ohmic behaviour)
	Glass	1.2×10^{-7}	2.74	1.41×10^{-9}
	Glass post water vapour exposure	8.5×10^{-9}	2.83	2.49×10^{-9}
[TPrA][Fe(dca)$_3$]	Crystalline	6.4×10^{-7}	7.41	8.03×10^{-7}
	Glass	-	-	1.71×10^{-10}
	Glass post water vapour exposure	-	-	1.21×10^{-11}
[TBuA][Mn(dca)$_3$]	Crystalline	4×10^{-8}	7.86	1.54×10^{-7}
	Glass	-	-	6.37×10^{-10}
	Glass post water vapour exposure	-	-	1.57×10^{-11}
[TBuA][Fe(dca)$_3$]	Crystalline	1.2×10^{-6}	0.64	1.01×10^{-6}
	Glass	4.2×10^{-9}	3.24	7.46×10^{-9}
	Glass post water vapour exposure	-	-	3.41×10^{-9}
[TPnA][Mn(dca)$_3$]	Crystalline	1.1×10^{-5}	1.59	7.59×10^{-7}
	Glass	-	-	1.54×10^{-10}
	Glass post water vapour exposure	-	-	8.76×10^{-11}
[TPnA][Fe(dca)$_3$]	Crystalline	4.8×10^{-5}	1.08	7.18×10^{-7}
	Glass	2.9×10^{-6}	0.62	5.22×10^{-8}
	Glass post water vapour exposure	1.2×10^{-6}	0.48	1.48×10^{-13}

CHN elemental analysis

Table S2 *CHN elemental analysis of the pre-WV and post-WV crystalline HOIPs.*

		[TPrA][Mn(dca)$_3$]	[TPrA][Fe(dca)$_3$]	[TBuA][Mn(dca)$_3$]	[TBuA][Fe(dca)$_3$]	[TPnA][Mn(dca)$_3$]	[TPnA][Fe(dca)$_3$]
%C	pre exposure	47.85	40.58	52.63	45.39	53.01	51.91
	post exposure	47.54	39.49	52.53	44.99	54.4	51.99
%H	pre exposure	6.25	5.6	7.28	6.41	7.91	7.49
	post exposure	6.2	5.4	7.18	6.3	8.1	7.48
%N	pre exposure	31.13	26.01	27.86	24.33	20.49	23.6
	post exposure	31.18	25.45	27.78	23.98	21.17	23.7

References

1 L. N. McHugh, M. F. Thorne, A. M. Chester, M. Etter, K. Uzarevic and T. D. Bennett, *Chem. Commun.*, 2022, **58**, 3949–3952.

2 Alan Coelho, TOPAS-Academic V7 Coelho Software 2020.

3 TA Instruments, Trios Software TA Instruments - Waters LLC 2020.

4 Micromeritics Instrument Corporation, Flex (version 6.02) 2022.

5 J. A. Schlueter, J. L. Manson and U. Geiser, *Inorg. Chem.*, 2005, **44**, 3194–3202.

6 J. M. Bermúdez-García, M. Sánchez-Andújar, S. Yáñez-Vilar, S. Castro-García, R. Artiaga, J. López-Beceiro, L. Botana, A. Alegría and M. A. Señarís-Rodríguez, *Journal of Materials Chemistry C*, 2016, **4**, 4889–4898.

7 B. K. Shaw, C. Castillo-Blas, M. F. Thorne, M. L. Ríos Gómez, T. Forrest, M. D. Lopez, P. A. Chater, L. N. McHugh, D. A. Keen and T. D. Bennett, *Chemical Science*, 2022, **13**, 2033–2042.

8 B. Yancey and S. Vyazovkin, *Phys. Chem. Chem. Phys.*, 2014, **16**, 11409–11416.

9 G. Han, L. M. Daniels, A. Vasylenko, K. A. Morrison, L. Corti, C. M. Collins, H. Niu, R. Chen, C. M. Roberston, F. Blanc, M. S. Dyer, J. B. Claridge and M. J. Rosseinsky, *Angewandte Chemie International Edition*, 2024, **63**, e202409372.
